# Combining positioning and labelling interventions for healthier and more environmentally sustainable products: A randomised controlled trial in an online experimental supermarket

**DOI:** 10.1016/j.appet.2025.108378

**Published:** 2025-11-06

**Authors:** Cinja Jostock, Alice O’Hagan, Rachel Pechey

**Affiliations:** Nuffield Department of Primary Care Health Sciences, University of Oxford, UK

**Keywords:** Food, Health, Sustainability, Positioning, Labels, RCT

## Abstract

Population diets need to become healthier and more sustainable to limit their negative effects on health and environment. This study assessed the effect of a positioning intervention, in isolation and in combination with a labelling intervention, on the a) healthiness and b) environmental sustainability of food choices in an experimental online supermarket. Participants (n = 2220) were randomly assigned to one of five trials groups (control; healthier items shown earlier (“health position”); health position & nutri-score labels; environmentally sustainable products shown earlier (“eco position”); eco position & ecolabels) and completed a shopping task in an experimental online supermarket. Linear regressions showed that compared to control, mean scaled nutri-scores of shopping baskets were significantly lower (healthier) for health position (−2.30; 95 %CI: −3.07, −1.52) and health position & labels (−2.50; 95 %CI: −3.28, −1.72), with no significant difference between health position and health position & labels (−0.20; 95 %CI: −0.66, 0.25). The mean eco scores of shopping baskets were significantly reduced (more sustainable) for eco position (−24 %; 95 %CIs: −15 %, −31 %) and eco position & labels (−30 %; 95 % CIs: −22 %, 37 %) compared to control. The eco position & labels group had significantly lower mean eco scores (−8 %; 95 % CIs: −2 %, −14 %) compared to eco position. The positioning intervention improved health and environmental sustainability of food selections in an experimental online supermarket, with less robust evidence for a small additional effect of adding labels. There was no suggestion that adding labels that potentially make the positioning intervention more salient had any backfire effects.

## Introduction

Unhealthy diets and climate change threaten population health; in 2021, dietary risks were the third highest risk factor contributing to premature death in the UK (Institute for Health Metrics and Evaluation, 2024), while the food system is a major source of global greenhouse gas emissions, contributing an estimated 34 % share (Crippa et al., 2021). Additionally, emerging evidence points to the adverse health impacts of climate change globally, with, for example, higher temperatures linked to cardiovascular and respiratory illnesses (Rocque et al., 2021). Thus, diets that are both healthier and more environmentally sustainable are urgently needed.

Online supermarkets are an increasingly popular choice for grocery shopping in the UK (Office for National Statistics, 2022), and offer a venue for potential intervention. One intervention strategy in food settings that has received increasing attention is targeting micro-environmental or choice architecture interventions (Hollands et al., 2013), such as altering product size, availability, or positioning, with meta-analyses suggesting these types of interventions are effective in influencing food selection, purchasing or consumption (Mertens et al., 2022). However, very few of these types of intervention have been assessed in combination (Jostock et al., 2025).

One promising type of micro-environmental interventions are positioning interventions, whereby target products are placed in more prominent locations (e.g. end-of-aisle, or where they are in closer proximity to individuals or may be seen earlier). Placement strategies are commonly used in online grocery stores and often target less healthy products (Maganja et al., 2024) Moran et al., 2022). A Cochrane review focusing on proximity interventions found (with very low and low certainty respectively) that decreased proximity of food items lowers their selection and consumption (Hollands et al., 2019). In addition, previous studies conducted in experimental online supermarkets have predominantly found that prominent positioning of products encouraged both more sustainable (Jostock et al., 2024) and healthier (Howe et al., 2022; Koutoukidis et al., 2019; Valenčič et al., 2023) food selections. While another online positioning study found no evidence of an effect, only six options were presented, all visible on the first page (Zhuo et al., 2023). Evidence suggests that almost all food choices in online supermarkets are made on the first page (Anesbury et al., 2016; Jostock et al., 2024), meaning re-positioning products across pages may be more effective than repositioning within the same page.

Labels are another tool that may encourage healthier or more environmentally sustainable food choices, with nutrition labels already widely implemented (Crockett et al., 2018; Pettigrew et al., 2023; Santé publique France, 2022), albeit more consistently applied to products in physical compared to online grocery stores (Bhatnagar et al., 2021). A Cochrane review suggests high certainty that calorie labelling influences food selection and purchasing, but that this effect is small (Clarke et al., 2025). This is consistent with other systematic reviews that have found positive (Cecchini & Warin, 2016; Song et al., 2021) or mixed and inconclusive (An et al., 2021) effects of front-of-pack nutrition labels on food selections or purchases. One popular front-of-pack label is the Nutri-Score label, used in seven European countries (Scientific Committee of the Nutri-Score, 2023) and found to be easily understandable (Egnell et al., 2018; Egnell et al., 2020 Shrestha et al., 2023; van den Akker et al., 2022), with most evidence suggesting that these labels encourage healthier intended and actual selections or purchases (De Temmerman et al., 2021; Egnell et al., 2022; Egnell et al., 2019; Song et al., 2021; van den Akker et al., 2022). Whilst not yet widely in use, studies in experimental online supermarkets found that environmental impact labels can be successful in increasing sustainable food choices (Muller et al., 2019; Potter et al., 2024). Additionally, labels appear to be a comparatively acceptable strategy to try to reduce meat consumption, receiving the lowest opposition (26 %) of six potential interventions in one study of public support (Pechey et al., 2022).

Interventions targeting health and environmental sustainability may not be equally effective. Evidence suggests that when it comes to food choice, health is deemed more important than environmental sustainability (Baudry et al., 2017; Blanke et al., 2022). Yet two systematic reviews found that while willingness to reduce meat intake for environmental reasons was low, informing participants about the environmental impact of meat production can increase willingness to reduce meat consumption (Hartmann & Siegrist, 2017; Sanchez-Sabate & Sabaté, 2019). Given that public understanding of the association between diet and health is relatively strong (Lappalainen et al., 1998; Paquette, 2005), while knowledge of the impact of diet on environmental sustainability is generally weaker (Sanchez-Sabate & Sabaté, 2019), ecolabels may be more likely to increase awareness of products’ environmental impact than health labels are to increase knowledge of products’ relative health impacts.

To the best of our knowledge, the impact of combining positioning and labelling interventions to encourage healthier or more environmentally sustainable food choices has not yet been investigated. In previous online supermarket positioning studies (Howe et al., 2022; Jostock et al., 2024; Koutoukidis et al., 2019; Valenčič et al., 2023; Zhuo et al., 2023), the environmental impact or healthiness of products was not indicated to participants and conveying this information may reinforce the impact of positioning interventions. However, labelling might also make positioning interventions more noticeable to consumers and thereby lead to unintended consequences (e.g. if an individual’s conscious decision-making counteracts non-conscious biases). Given the potential variation in the impact of environmental sustainability vs. health labelling, it is also important to explore the effects within each of these domains.

This study evaluated the impact of a positioning intervention, in isolation and in combination with a labelling intervention, on the a) healthiness and b) environmental sustainability of food choices in an experimental online supermarket. We assessed whether there was a difference in impact when the positioning intervention was combined with product information labels.

## 2 Methods

### 2.1 Study design

We conducted a randomised controlled trial with five trial groups, consisting of one control and four intervention groups (see Table 1). Participants completed the study in March and April 2024. We pre-registered the study protocol with the Open Science Framework (OSF) (osf.io/q68nm). Ethical approval for this study was obtained from the University of Oxford’s Medical Sciences Interdivisional Research Ethics Committee (IDREC) (ref: R65010/RE016).

#### 2.1.1 Study conditions

##### Control condition

**Fig. 1:**
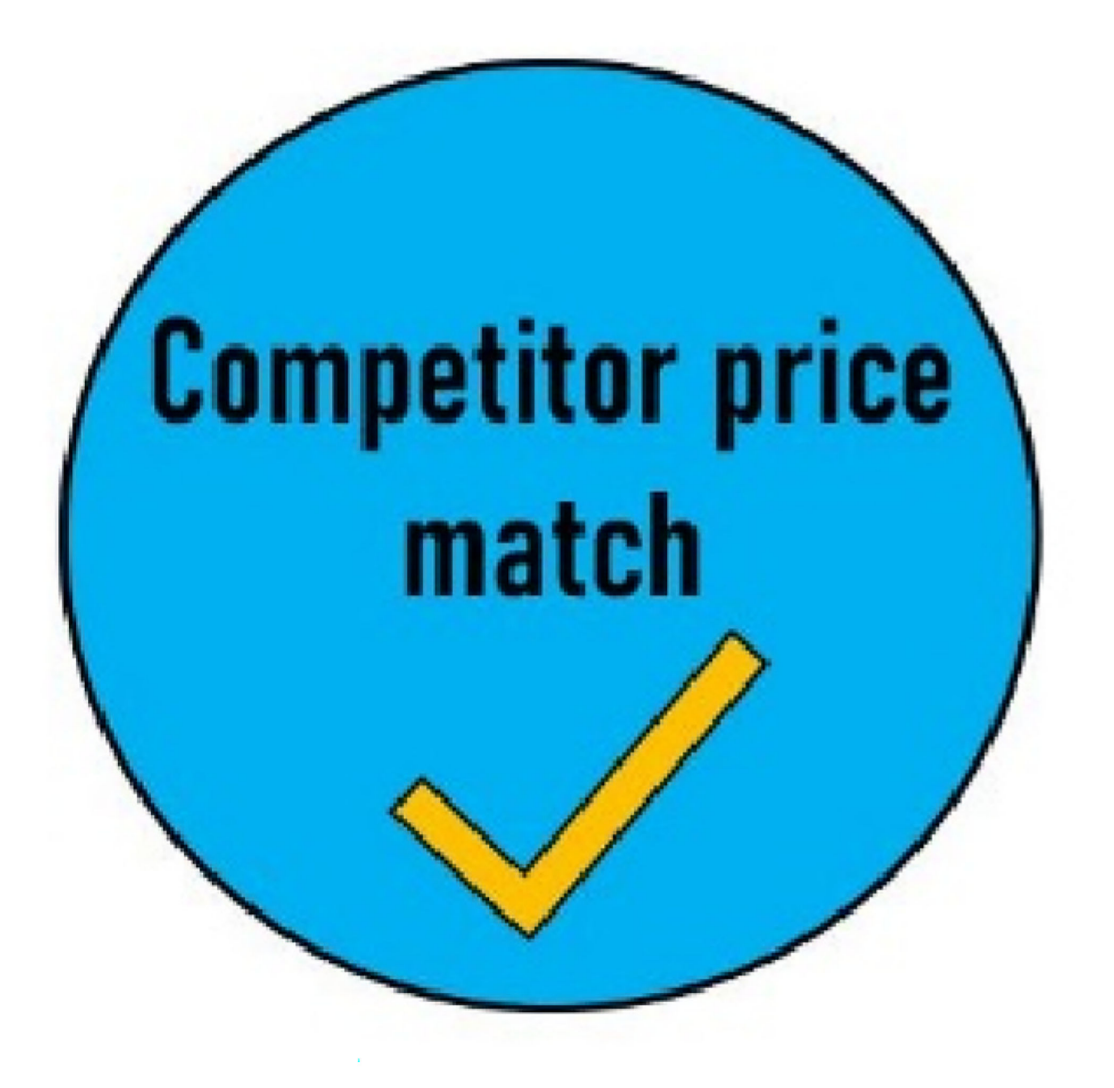
Price match label.

Products in the control condition were assigned a random number between 1 and 6 (e.g. 1.345835213; 3.204049279) and ordered ascendingly. We added a price match label (Fig. 1) for a random subset of 10 % of products to counteract the risk of an experiment demand bias when viewing labels on the experimental supermarket in the health and sustainability label conditions. No other labels were applied to products.

##### Health position

We biased the order in which products appeared in the supermarket to make healthier products more likely to show up in earlier positions. We biased the order of products as follows.

- Health label “A”: products assigned a random value between 1 and 2
- Health label “B”: products assigned a random value between 1.1 and 3
- Health label “C”: products assigned a random value between 1.2 and 4
- Health label “D”: products assigned a random value between 1.3 and 5
- Health label “E”: products assigned a random value between 1.4 and 6

The health labels of products were not shown in the supermarket for the health position only group. Price match labels were applied exactly as in the control group.

##### Eco position

We biased the order of products using the same procedure as in the health position group, but favouring more environmentally sustainable (henceforth “sustainable”) products over less sustainable products.

- Eco label “A”: products assigned a random value between 1 and 2.
- Eco label “B”: products assigned a random value between 1.1 and 3.
- Eco label “C”: products assigned a random value between 1.2 and 4.
- Eco label “D”: products assigned a random value between 1.3 and 5.
- Eco label “E”: products assigned a random value between 1.4 and 6.

No eco labels were displayed alongside products in the supermarket for the eco position only group. Again, price match labels were the same as in the control group.

##### Health position & labels

**Fig. 2:**
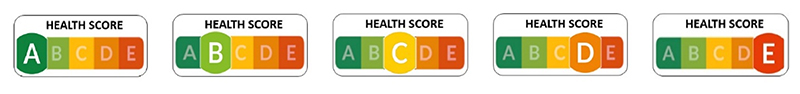
Health labels.

The same positioning intervention as in the health position condition was combined with health labels ranging from “A” to “E” (values for health scores were determined using the original Nutri-Score algorithm (Julia et al., 2017), scaled to range from 0 to 100; the label design was based on Nutri-Score; Fig. 2). “A” denotes products with the best health scores and “E” the ones performing worst in terms of healthiness. Price match labels were displayed in the same manner as in the other groups. No information was presented to participants about the health labels.

##### Eco position & labels

**Fig. 3:**
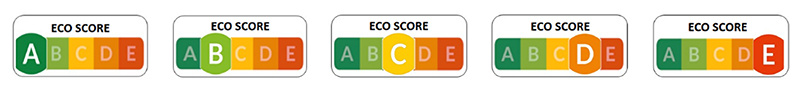
Eco labels.

In addition to the positioning intervention from the eco position group, eco labels indicating the environmental sustainability of products were applied, ranging from most sustainable “A” to least sustainable “E” (label design matching the health labels; Fig. 3). Sustainability was based on a composite score, considering data on greenhouse gas emissions, scarcity-weighted water use, biodiversity loss and eutrophication (Clark et al., 2022). Scores (“eco scores”) were scaled to range from 0 to 100., with ecolabel scores assigned to products based on their eco score quintile. Price match labels were applied as in all other groups. No information was presented to participants about the eco labels.

### 2.2 Setting

**Fig. 4:**
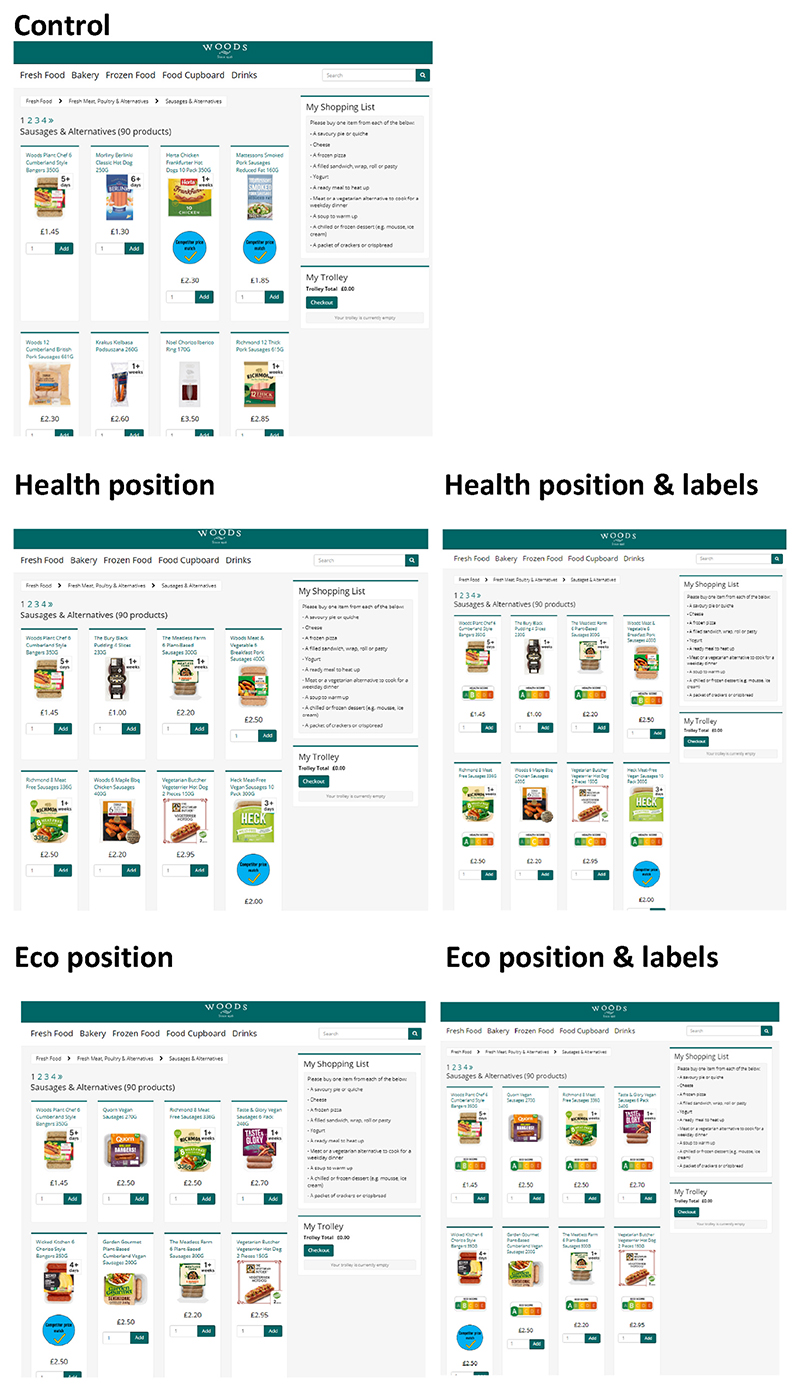
Example online supermarket page of a product shelf (Sausages & Alternatives) in each group.

The shopping task was completed in an experimental online super-market (www.woodssupermarket.co.uk); see Fig. 4 for an example of how the first eight products on one target category shelf were displayed for each trial group.

Around 8400 products from the largest UK retailer were available for selection in the supermarket. We used product data from the foodDB database, which collects information about food and drink products offered in UK online supermarkets (Harrington et al., 2019). We removed alcoholic products and no-or-low alcohol equivalents from the dataset because Nutri-Score does not apply to alcoholic beverages (Scientific Committee of the Nutri-Score, 2023) and our study focus was on food. We also removed products with incomplete nutrient information for our nutrients of interest (i.e. energy (kcal), salt, sugar, fat).

Each page in the online supermarket can display a maximum of 28 products; if more than 28 items are allocated to a shelf, subsequent pages are created. Products can be searched using a drop-down menu with departments, aisles and shelves, or, alternatively, using a search bar. We ordered the departments, aisles and shelves as similar to the UK retailer as possible. However, we made some modifications to aisles and shelves to increase the strength and salience of the positioning intervention. For example, instead of having a separate shelf for vegetarian and plant-based products, we sorted these into meat aisles so that they would be displayed alongside and directly compete with meat products. We also combined some of the shelves so that most shelves consisted of several pages, as positioning interventions appear to be more effective if increased scrolling is required to reach dis-incentivised products (Jostock et al., 2024) (e.g., summarising cheese shelves into “All Cheese”, “Grated, Sliced & Block Cheese”, “Cottage Cheese & Soft Cheese”, “Counter Cheese”, and “Cheese Snacks”).

### 2.3 Study procedure

The study procedure included: a screening survey assessing informed consent and eligibility criteria; a baseline survey asking about demographics, shopping, dietary and health data (including meat consumption, use of online supermarkets, weekly spend on groceries, and perceptions of the importance of healthy and/or sustainable diets; see Table 2); the shopping task in the online experimental supermarket; and a post-intervention survey assessing the acceptability of the interventions as well as usual shopping behaviours. Surveys were conducted using Qualtrics (see Appendix A for questions).

#### Shopping task

Participants were given the following instructions for the task:

“We would like you to do some online grocery shopping on a supermarket website. This is not a real supermarket, and you will not be asked to spend any of your own money.

**We will give you a shopping list and ask you to select a food item to match each item on the list, which will be displayed on the right hand side of the screen (see picture below)**.

**Please do not select additional items**.

When doing your shopping, try to imagine you are doing your own grocery shopping and choose foods that you would eat. You should choose the things you normally eat or would be willing to eat as far as possible. Do not choose any foods that you would not be willing to eat.

**Figure:**
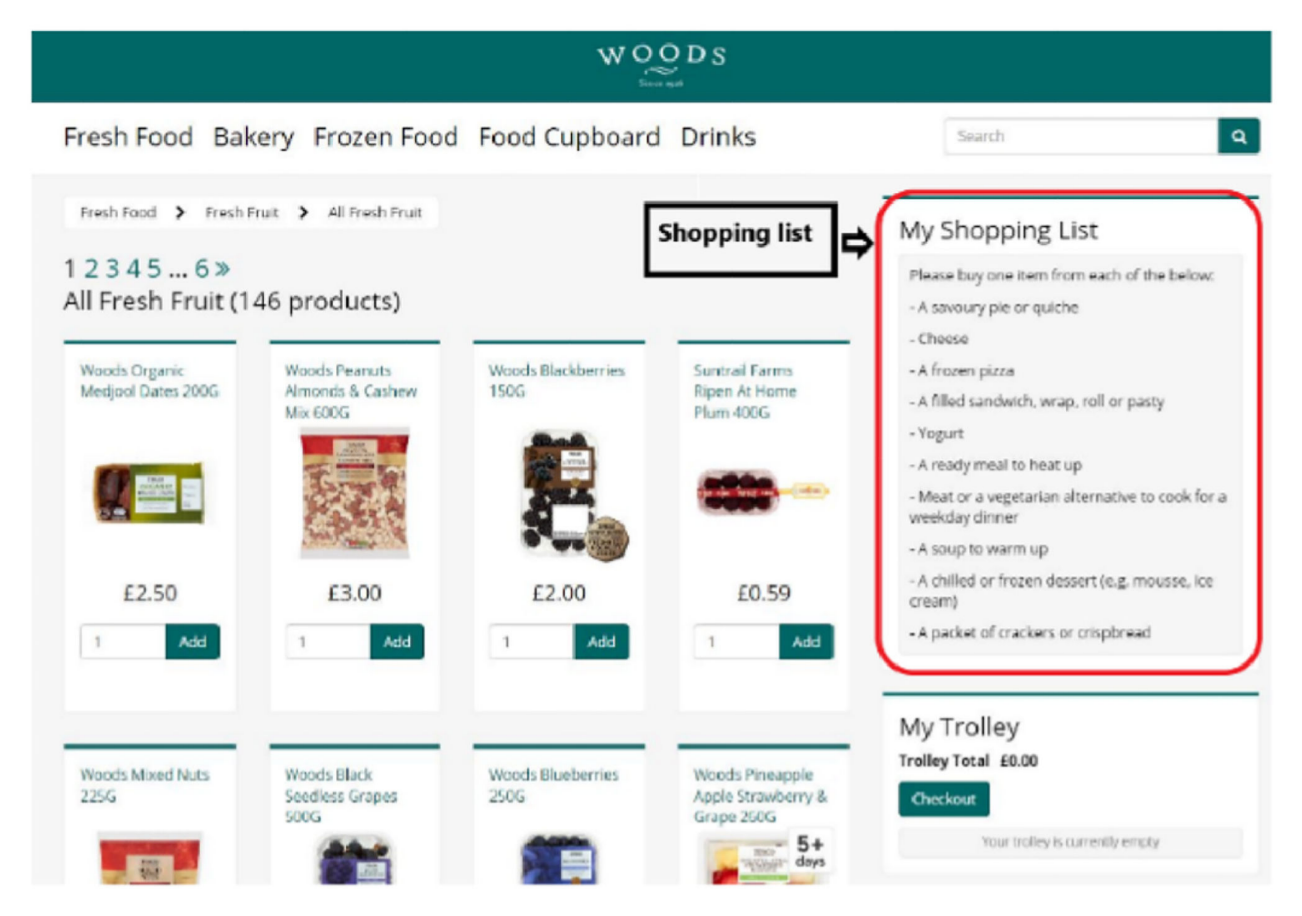

”

The shopping list was shown on the right hand side of the online supermarket web page as a reminder for participants. Participants were instructed to select a product for the following categories.

- *A savoury pie or quiche*
- *Cheese*
- *A frozen pizza*
- *A filled sandwich, wrap, roll or pasty*
- *Yogurt*
- *A ready meal to heat up*
- *Meat or a vegetarian alternative to cook for a weekday dinner*
- *A soup to warm up*
- *A chilled or frozen dessert* (e.g. *mousse, ice cream)*
- *A packet of crackers or crispbread*

To check out of the supermarket, participants had to select a minimum of 10 and a maximum of 15 different products. The shopping list was adapted from one used in a previous study (focused only on sustainability) (Jostock et al., 2024), with items selected that had variation in health and/or sustainability in our dataset.

### 2.4 Participants and randomisation

We aimed to recruit participants broadly representative of the UK in terms of gender, age, and education. Participants were recruited through Dynata (https://www.dynata.com) and had to be aged 18 or over, live in the UK, be able to speak and read English, be able to provide informed consent, and have access to the internet and a device that can access the internet (e.g. computer, tablet, mobile phone).

We assumed a medium effect size (f = 0.25) for the positioning intervention (Jostock et al., 2024) and a small effect size (f = 0.1) for the add-on effect of the labelling intervention (with a power 0.9, and alpha of 0.05). We factored in a 20 % non-compliance rate based on a similar previous study (Jostock et al., 2024), giving a sample size of 2659 participants, with around 127 participants in the control group and 633 for each intervention group.

At the end of the baseline survey, Qualtrics randomised the participants into one of the five groups on a 1:5:5:5:5 basis, so that numbers allocated to the intervention groups would outweigh the control group by a 5:1 ratio, to account for the smaller expected effect size for between intervention comparisons.

### 2.5 Primary analyses

All analyses were run in RStudio (Posit team, 2024; R Core Team, 2022), using dplyr (Wickham, François, et al., 2023), tidyr (Wickham, Vaughan, & Girlich, 2023), rstatix (Kassambara, 2023), car (Fox & Weisberg, 2019), ggplot2 (Wickham, 2016), table1 (Rich, 2023), and broom (Robinson et al., 2022) packages.

We excluded from analyses participants who met any of the following criteria: Did not complete the full study; Selected more than 20 products; Finished the study in less than 30 % of the median time (“Speeder”); Did not comply with the shopping task, with non-compliance defined as selecting items for fewer than 7 of the required product categories. Sensitivity analyses excluded observations that fell above Q3 + 1.5xIQR or below Q1 - 1.5xIQR.

The same methods were used to evaluate results for a) healthiness (3 study conditions included in the analysis: control, health position, and health position & labels) and b) sustainability (3 study conditions included in the analysis: control, eco position, and eco position & labels).

The primary outcomes were the a) basket mean scaled Nutri- Scores (hereafter Nutri-Score) and b) basket mean scaled eco scores (which was logged for analyses) (hereafter eco score). Higher scores indicate less healthy and less sustainable foods. Scores were compared using linear regression models, with the control group as the reference group and a significance threshold of p < 0.025. Additionally, separate models compared the position group (reference) to the position & labels group for both (i) health and (ii) sustainability to investigate a potential add-on effect of labels.

#### Sensitivity analyses

The first sensitivity analysis for both a) healthiness and b) sustainability groups included participants with full compliance to the shopping task. Full compliance was met if participants selected one product for each shopping list category and no additional items. The same models and significance level as in the primary analysis were applied.

During analyses, we noticed that a small number of products appeared to have incorrect Nutri-Scores. Further exploration of products’ nutrition data suggested that this may have been due to 326 products having commas instead of dots to indicate decimals in the nutrient information. Where possible we recalculated the Nutri-Score using correct nutrition information, of these products 40 % did not change in their label value, 29 % were out by one letter (e.g. B rather than C), 20 % by two letters, 7 % by 3 and 4 % by 4. We conducted additional sensitivity analyses removing all affected products that were selected by one or more participants (n = 222) as well as participants who selected affected products in the health position & labels group (n = 90; 17 % of participants).

### 2.6 Secondary analyses

#### Nutrients and environmental indicators

To obtain more detailed insights into the effect of the interventions, we assessed the difference in a) mean energy density (kcal/100g or kcal/100 ml), mean salt content (g/100g), mean fat content (g/100g), and mean sugar (g/100g) content of shopping baskets and b) basket mean environmental indicator scores (logged) (namely: i) greenhouse gas emissions (kg CO_2_e), ii) water use (litres), iii) biodiversity loss (species lost x 10^−14^) and iv) eutrophication potential $({\text{gPO}}_{4}^{3-}\text{e}),$ each per 100g of product). Linear regression models included: a) health groups vs. control (reference) and b) sustainability groups vs. control (reference). We applied a significance threshold of p < 0.005 (Bonferroni adjustment).

The mean eco score and mean environmental indicator scores of shopping baskets were log-transformed due to skewness for all regression analyses using these outcome measures. Intercepts, coefficients and confidence intervals of models were exponentiated to facilitate interpretation of regression results.

For fat, salt, and sugar data, we recoded any mentioning of “trace”, “nil”, and “negligible” amounts as zero (Department of Health, 2017). For fat and sugar, any entries under 0.5 were recoded as zero, whilst for salt any entries smaller than 0.01 were recoded as zero (Department of Health, 2017) and “<” was removed from any values higher than 0.01 to allow for aggregation (e.g. <0.02 was changed to 0.02).

#### Interactions by participant characteristics

We evaluated if the effect of the interventions was modified by participant characteristics (gender, age, education, income, and meat consumption) to assess potential equity impacts. Multiple linear regression models had a significance level of p < 0.005 (Bonferroni adjustment).

We measured meat and dairy consumption through three questions assessing the frequency of meat and dairy consumption at breakfast, lunch, and dinner. Aggregated meat and dairy consumption scores were calculated by summing points assigned to answers: 0 for "Never", 1 for "less than once a week", 2 for "1–2 days a week", 3 for "3–4 days a week", 4 for "5–6 days a week", and 5 for "Every day". Then, three levels were determined: "low" (overall scores 0–5), "medium" (scores 6–10), and "high" (scores 11–15).

#### Basket price

We assessed whether the interventions affect the total price (£GBP) of shopping baskets through descriptive statistics and ANOVA tests for a) healthiness and b) sustainability, with a significance level of p < 0.025 (Bonferroni adjustment).

#### Deviations from protocol

Due to an error, our pre-registration stated that we would use a p-value threshold of 0.05 for comparisons between Position and Position & Health groups, rather than 0.025 as with our other primary analyses, and following Bonferroni adjustment for the number of comparisons; the value of 0.025 is used here. Although in our protocol we planned on using more sub-groups for the gender, age group, income and education variables, we decided to combine adjacent groups to achieve bigger group sizes (Appendix B). Additionally, due to small group sizes, participants who indicated “other gender identity” were not included in models and those who selected “prefer not to say” when asked about their income were excluded from income interaction models. Participants for whom we could not calculate a meat consumption score due to missing data were also dropped from models.

### 2.7 Exploratory analyses

#### Spill-over effects

We wanted to explore whether interventions aimed at health would also lead to more sustainable product selections and vice versa. We ran the same models as in the primary analysis, but changed the outcome for the model investigating the health groups to mean eco scores of shopping baskets and the model assessing the sustainability groups to the mean Nutri-Scores of shopping baskets.

#### Acceptability of the interventions

To test whether the interventions were acceptable to participants, we included three acceptability questions in the post-intervention survey using 7-point scales (Appendix C). We evaluated responses using descriptive statistics.

#### Interactions by health or environmental consciousness

We wanted to investigate whether the intervention effect differed by health and environmental consciousness, which was measured through two questions in the post-intervention survey using 5-point scales (Appendix C). The coding of health and environmental consciousness is provided in Appendix B. We evaluated differences in a) mean Nutri-Scores by health consciousness and b) eco scores by environmental consciousness through multiple linear regression models with interaction terms.

## Results

**Fig. 5:**
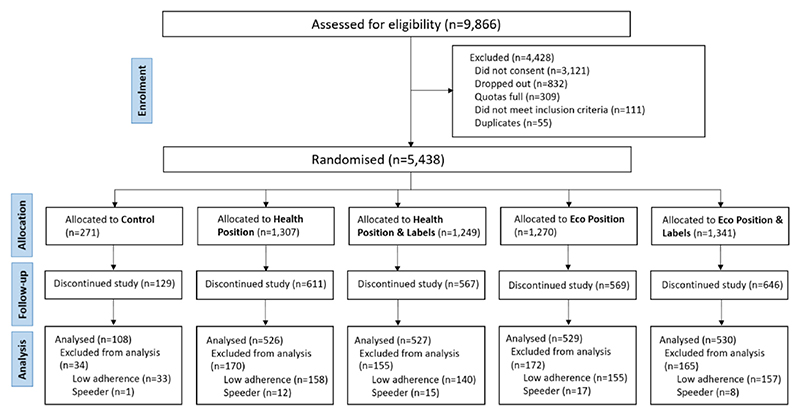
CONSORT diagram (Schulz et al., 2010).

We encountered technical problems with our online supermarket platform, which resulted in high dropout between randomisation and study completion (Fig. 5). A total of 2916 participants completed the study. After data exclusions, mostly due to low adherence to the shopping task, 2220 participants were included in the analysis (Fig. 5).

Participants were a mean age of 48 years (range: 18–76 years) and lived in households with a median size of 2 persons (Table 2). Overall, participants reported spending a mean of £95.89 on their weekly food shop(s). About two-thirds of participants reported shopping online for food at least once in the last year. Around 7 % of the sample reported following a vegetarian or vegan diet.

### 3.1 Primary analyses

#### 3.1.1 Health

The mean Nutri-Scores of shopping baskets were highest in the control group (35.7), followed by the health position (33.4) and the health position & labels (33.2) groups, with higher mean Nutri-Scores indicating less healthy product selections. Table 3 provides the means and standard deviations for the health and sustainability outcomes, for each study condition.

Compared to control, mean Nutri-Scores of shopping baskets were significantly lower in the health position (−2.30; 95 %CI: −3.07, −1.52) and the health position & labels (− 2.50; 95 %CI: −3.28, −1.72) group (Table 4). However, there was no significant difference in the mean Nutri-Scores of shopping baskets between the health position and health position & labels (− 0.20; 95 %CI: −0.66, 0.25) group.

##### Sensitivity analysis (full compliance sample)

Similar to the primary analysis, mean Nutri-Score of shopping baskets was significantly reduced in the health position and health position & labels groups compared to control, with a slightly increased effect size (see Table 4).

##### Sensitivity analysis removing products affected by the decimal error and purchasers of these products

Results were similar to the main analysis (i. e., health position and health position & labels significantly reduced Nutri-Scores compared to control, and there was no significant difference between health position and health position & labels) (Appendix D).

#### 3.1.2 Sustainability

The mean eco score of shopping baskets was highest in the control group (4.67), followed by the eco position (3.68), and lowest in the eco position & labels group (3.40) (Table 3).

Compared to the control group, the mean eco score of shopping baskets was significantly reduced in the eco position (− 24 %; 95 %CIs: −15 %, −31 %) and the eco position & labels group (−30 %; 95 % CIs: −22 %, 37 %) (Table 4). The addition of labels in the eco position & labels group significantly reduced the mean eco score of shopping baskets in this group (− 8 %; 95 % CIs: −2 %, −14 %) compared to the eco position group.

##### Sensitivity analysis (*full compliance sample*)

As in the primary analyses, the mean eco scores of shopping baskets were significantly lower in the eco position and eco position & labels groups compared to control (see Table 4). However, whilst the effect size remained the same when comparing shopping baskets in the eco position & labels group and in the eco position group in the primary and sensitivity analyses, this did not reach significance in the smaller sample sensitivity analysis (8 % decrease; 95 % CIs: 16 % decrease; 1 % increase).

### 3.2 Secondary outcomes

#### 3.2.1 Nutrients

Shopping baskets of participants from the health position and the health position & labels group had a lower mean salt and fat content compared to control, with the health position & labels group also having a significantly reduced mean energy content (see Table 5). However, in sensitivity analyses when outliers were removed, the difference in mean salt content of shopping baskets between the control and health position group was no longer significant (Appendix E). No significant differences in mean sugar content of shopping baskets were found between the intervention groups and control.

#### 3.2.2 Environmental indicators

Shopping baskets in the eco position and the eco position & labels group had significantly lower mean greenhouse gas emissions (eco position: −24 % (95 %CIs: −15 %,-32 %); eco position & labels: −31 % (95 %CIs: −23 %,-39 %)), biodiversity loss (eco position: −21 % (95 %CIs: −11 %,-30 %); eco position & labels: −28 % (95 %CIs: −19 %,-36 %)), water use (eco position: −25 % (95 %CIs: −19 %,-31 %); eco position & labels: −29 % (95 %CIs: −24 %,-34 %)), and eutrophication (eco position: −22 % (95 %CIs: −9 %,-33 %); eco position & labels: −30 % (95 % CIs: −19 %,-40 %)) compared to control (see Table 6).

#### 3.2.3 Interactions by demographic characteristics

There were no significant interactions by gender, age group, education, income, or meat consumption for either health or sustainability models. Models are contained in Appendix F (health) and G (sustainability). Exploratory analyses by health or environmental consciousness also showed no interaction (Appendix H). Adjusted models comparing positioning & label groups to positioning only can be found in Appendix I.

### 3.3 Price

#### 3.3.1 Health

The mean summed basket price was highest in the control group (GBP £24.09), followed by health position (£22.78) and lowest in the health position & labels group (£22.45) (see Appendix J for means and SDs of each secondary outcome by study condition). The price difference between the health position & labels groups and control was significant; but not for health position and control (Table 5). No difference was observed between intervention groups (−0.33; 95 %CIs: −1.10, 0.44; p = 0.570).

#### 3.3.2 Sustainability

The control group had the highest mean basket price (GBP £24.09), followed by the eco position (£22.48) and eco position & labels group (£22.33). Compared to control, there were significant reductions in the summed basket prices in the eco position and eco position & labels groups (Table 6). There was no significant difference between the intervention groups (− 0.15; 95 %CIs: −0.85, 0.55; p = 0.875). However, when outliers were removed, although the mean price was still highest for shopping baskets in the control group, the differences between groups were not significant (Appendix K).

### 3.4 Exploratory outcomes

#### 3.4.1 Spill-over effects

##### 3.4.1.1.Health

There was no evidence for differences in the mean eco score of products in shopping baskets between the control and the health intervention groups (Table 5).

##### 3.4.1.2 Sustainability

Analyses suggested shopping baskets in the eco position and eco position & labels groups may have had higher mean Nutri-Scores (i.e. lower healthiness) than control (Table 6). The difference between eco position & labels and control was no longer significant with outliers removed (Appendix L).

#### 3.4.2 Acceptability

**Fig. 6:**
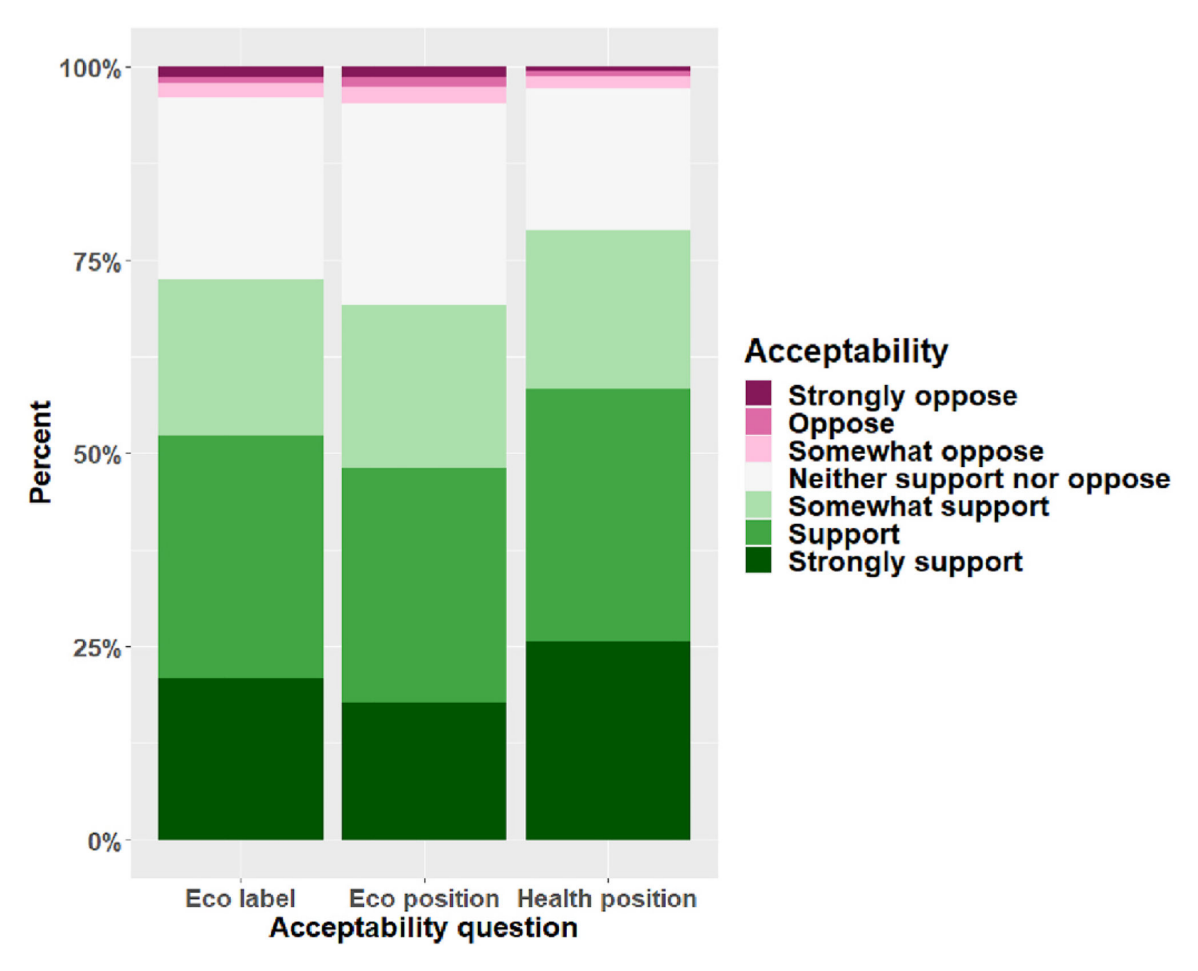
Acceptability of the positioning and eco labelling intervention. *Note*. We asked the following questions: *Eco label:* “If supermarkets were to label products to indicate their environmental impact, to what extent would you support or oppose this?”; *Eco position:* “If supermarkets were to position products to emphasise more environmentally sustainable products, to what extent would you support or oppose this?”; *Health position:* “If supermarkets were to position products to emphasise healthier products, to what extent would you support or oppose this?”

The interventions were supported by the majority of participants (Fig. 6). Although all interventions were overall acceptable, the health positioning intervention was rated as most acceptable, with 78.8 % of participants supporting this intervention to any extent, compared to 72.5 % for eco labels and 69.2 % for eco position.

As health labels are already widely implemented, we did not ask about their acceptability but rather current usage of front-of-pack nutrition labels when shopping: 41.4 % of participants reporting using such labels always or often, 34.3 % reporting using them sometimes, and 24.4 % said they rarely or never used such labels.

## 4 Discussion

Positioning products to favour healthier or more sustainable products improved the healthiness and environmental sustainability of shopping baskets respectively in an experimental online supermarket. There was some evidence for a small additional effect of adding sustainability labels to the positioning intervention, although this was less robust. Whilst we found reductions in the environmental impact of shopping baskets with sustainability labels compared to positioning alone in the main analysis, this effect dropped out of statistical significance in the sensitivity analysis, with the reduced sample size one potential reason for this. There was no evidence that intervention effects differed by demographic factors.

Shopping baskets in the sustainability intervention groups had lower mean environmental indicator scores (i.e. greenhouse gas emissions, biodiversity loss, water scarcity and eutrophication) compared to control. Results were somewhat less robust for nutrients in the health groups: whilst fat, energy and salt content of shopping baskets were lower in both intervention groups, these were not significant for all comparisons. We found no differences in the sugar content of shopping baskets. In general though these results reflect that the interventions were successful in altering the outcomes of consequence for population and planetary health.

This RCT used realistic interventions in a naturalistic supermarket, with a large sample with characteristics broadly representative of the UK. The health and eco labels looked similar to the Nutri-Score design which is already used in seven European countries (although not in the UK) (Santé publique France, 2022). The main limitation of our study is its hypothetical nature, with participants not actually paying for or receiving any of the products they select, though results are in line with in-person studies identified in Cochrane reviews, which suggested a moderate effect of positioning and a very small effect of labelling (Clarke et al., 2025; Hollands et al., 2019). Additionally, due to technical issues with the Woods website (crashing under high participant volumes), we encountered high dropout rates, which risked introducing bias in terms of participant representativeness, although our sample characteristics broadly reflect the UK population. In the label groups, participants may have been able to guess the study purpose and adjusted behaviour in accordance with social desirability bias. However, to minimise this risk and distract participants from the health and eco labels, we displayed additional price match labels for the same random subset of products in all trial groups. In addition, the error with the nutri-labels on a small proportion of products could potentially have impacted the credibility of these labels – however, this is thought to have likely minimal impact given 0.9 % of the total products were out by more than one letter, and sensitivity analyses did not suggest differences. Finally, as participants may have been familiar with nutri-labels, but not eco labels, participants may have engaged to a different degree with these labels, or even mistaken the ecolabels for nutri-labels, which may not reflect longer-term behaviour should these be implemented.

The effectiveness of positioning interventions is in line with most previous online experimental supermarket studies that tested such interventions (Howe et al., 2022; Jostock et al., 2024; Koutoukidis et al., 2019; Valenčič et al., 2023), with the one previous study that found no effect offering a much lower number of products compared to our study, which may negate the impact of this intervention as all options may be visible (Zhuo et al., 2023). Our findings on labelling are also in line with the Cochrane review showing a small effect (Clarke et al., 2025). In our study, the mixed evidence found could reflect a very small effect that we may have been underpowered to detect. It could also potentially be exacerbated if the positioning intervention reduced the variation in label scores on the first page – however, data checks suggested that on the whole the range available (e.g. A to E) only changed to a small extent, although there were more options for healthier/more sustainable values and fewer for the less healthy/sustainable values. Importantly, there was no suggestion that adding labels that potentially make the positioning intervention more salient had any backfire effects, for example, with people showing reactance to the interventions.

There were no interactions by demographic characteristics or health or environmental consciousness, although these were potentially underpowered, suggesting that the interventions may be effective regardless of demographic characteristics or the perceived importance of healthiness and sustainability of foods. Additionally, the mean price of shopping baskets was lower in all intervention groups compared to control, although the difference was not always significant. Nevertheless, this demonstrates the affordability of the interventions for consumers, with no evidence that either intervention could have adverse effects on health inequalities.

Whilst the health interventions seemed to have no impact on the sustainability of products in shopping baskets compared to control, we found a trend for shopping baskets in the sustainability position and position & labels groups to be less healthy than in the control group. Although there is a correlation between the healthiness and sustainability of foods (Clark et al., 2022), this highlights that more sustainable does not necessarily mean healthier. While this may in part be an arte-fact of the particular categories selected here - by chance items that are sustainable but not healthier may have become more salient in our positioning interventions – it emphasises the need to plan interventions across both dimensions rather than assuming a complementary outcome.

We found that the positioning interventions and the sustainability label intervention were acceptable, with a clear majority of participants supportive of such measures. The health position intervention had the highest support, which is in line with our finding that participants perceive health as more important than sustainability when it comes to selecting food. Previous evidence similarly shows the higher importance attached to healthiness over sustainability of foods (Baudry et al., 2017; Blanke et al., 2022). Additionally, the sustainability labelling intervention received slightly higher support than the sustainability position intervention, which corresponds to existing evidence of a preference for less intrusive interventions (Pechey et al., 2022).

We tested the effect of positioning and label interventions in a controlled environment (i.e. experimental online supermarket), demonstrating that these can be used together without detriment, adding to a limited evidence base when it comes to combining intervention types. In real-world online supermarkets, multiple marketing strategies are usually employed concurrently (Khandpur et al., 2020; Moran et al., 2022), and both labelling and positioning have been the focus of recent policy attention. While Nutri-Score use is voluntary, calorie labelling is mandatory for larger businesses in both England and the US. Positioning interventions are also being introduced, such as the restrictions in England on the placement of high fat, sugar and/or salt products in prominent locations, with healthy checkout policies also implemented in California. The recent announcement in the UK that large food businesses will be required to report on the healthiness of their food sales, with the aim of implementing healthier sales targets in the future (UK Government, 2025), highlights the need to identify interventions that can be implemented effectively simultaneously.

## 5 Conclusion

The positioning interventions improved health and environmental sustainability of food selections in an experimental online supermarket, with less robust evidence for a small additional effect of adding labels. There was no suggestion that adding labels, which could potentially make the positioning intervention more salient, had any backfire effects.

## Supplementary Material

**Appendices. Supplementary data**

Supplementary data to this article can be found online at https://doi.org/10.1016/j.appet.2025.108378.

Supplementary information

## Figures and Tables

**Table 1:** Target foods and experimental manipulations in the control & intervention conditions.

	Control	Health position	Eco position	Health label & position	Eco label & position
*Price match label*	**X**	**X**	**X**	**X**	**X**
*Position*		**♦**	**▲**	**♦**	**▲**
*Health label*				**♦**	
*Eco label*					**▲**

♦ = intervention targeted at healthier food choices ▲ = intervention targeted at more environmentally sustainable food choices

**Table 2:** Demographics, shopping habits and health and environmental consciousness of the study sample.

	Control		Health Position		Health Position & Labels		Eco Position		Eco Position & Labels		Overall
	(N = 108)		(N = 526)		(N = 527)		(N = 529)		(N = 530)		(N = 2220)
**Gender identity**											
Female	62 (57.4 %)		282 (53.6 %)		263 (49.9 %)		266 (50.3 %)		268 (50.6 %)		1141 (51.4 %)
Male	46 (42.6 %)		244 (46.4 %)		260 (49.3 %)		262 (49.5 %)		260 (49.1 %)		1072 (48.3 %)
Other gender identity	0 (0 %)		0 (0 %)		4 (0.8 %)		1 (0.2 %)		2 (0.4 %)		7 (0.3 %)
**Age group**											
18-24	10 (9.3 %)		53 (10.1 %)		60 (11.4 %)		51 (9.6 %)		60 (11.3 %)		234 (10.5 %)
25-34	15 (13.9 %)		81 (15.4 %)		83 (15.7 %)		94 (17.8 %)		91 (17.2 %)		364 (16.4 %)
35-44	22 (20.4 %)		78 (14.8 %)		80 (15.2 %)		86 (16.3 %)		97 (18.3 %)		363 (16.4 %)
45-54	15 (13.9 %)		99 (18.8 %)		95 (18.0 %)		76 (14.4 %)		85 (16.0 %)		370 (16.7 %)
55-64	13 (12.0 %)		100 (19.0 %)		89 (16.9 %)		85 (16.1 %)		79 (14.9 %)		366 (16.5 %)
65+	33 (30.6 %)		115 (21.9 %)		120 (22.8 %)		137 (25.9 %)		118 (22.3 %)		523 (23.6 %)
**Highest level of education**											
No qualifications	8 (7.4 %)		21 (4.0 %)		34 (6.5 %)		32 (6.0 %)		37 (7.0 %)		132 (5.9 %)
Up to 4 GCSE’s	16 (14.8 %)		89 (16.9 %)		98 (18.6 %)		86 (16.3 %)		73 (13.8 %)		362 (16.3 %)
5 or more GCSE’s/1 A-level	18 (16.7 %)		112 (21.3 %)		106 (20.1 %)		110 (20.8 %)		119 (22.5 %)		465 (20.9 %)
2 or more A-levels	24 (22.2 %)		97 (18.4 %)		100 (19.0 %)		104 (19.7 %)		118 (22.3 %)		443 (20.0 %)
Bachelor’s degree or above	42 (38.9 %)		207 (39.4 %)		189 (35.9 %)		197 (37.2 %)		183 (34.5 %)		818 (36.8 %)
**Household income**											
Below £15.5K	10 (9.3 %)		73 (13.9 %)		78 (14.8 %)		86 (16.3 %)		69 (13.0 %)		316 (14.2 %)
Between £15.5K up to and including £25K	23 (21.3 %)		95 (18.1 %)		94 (17.8 %)		85 (16.1 %)		101 (19.1 %)		398 (17.9 %)
Between £25K and £39K	28 (25.9 %)		123 (23.4 %)		133 (25.2 %)		131 (24.8 %)		126 (23.8 %)		541 (24.4 %)
£40K or above	42 (38.9 %)		211 (40.1 %)		201 (38.1 %)		201 (38.0 %)		216 (40.8 %)		871 (39.2 %)
Prefer not to say	5 (4.6 %)		24 (4.6 %)		21 (4.0 %)		26 (4.9 %)		18 (3.4 %)		94 (4.2 %)
**Meat consumption**											
Low	27 (25.0 %)		154 (29.3 %)		138 (26.2 %)		138 (26.1 %)		141 (26.6 %)		598 (26.9 %)
Medium	56 (51.9 %)		247 (47.0 %)		240 (45.5 %)		238 (45.0 %)		223 (42.1 %)		1004 (45.2 %)
High	25 (23.1 %)		124 (23.6 %)		148 (28.1 %)		153 (28.9 %)		164 (30.9 %)		614 (27.7 %)
Missing	0 (0 %)		1 (0.2 %)		1 (0.2 %)		0 (0 %)		2 (0.4 %)		4 (0.2 %)
**Following vegetarian or vegan diet**											
No	101 (93.5 %)		482 (91.6 %)		499 (94.7 %)		495 (93.6 %)		483 (91.1 %)		2060 (92.8 %)
Yes	7 (6.5 %)		44 (8.4 %)		28 (5.3 %)		34 (6.4 %)		47 (8.9 %)		160 (7.2 %)
**Online shopping in the past year**											
Never or not in the last year	34 (31.5 %)		179 (34.0 %)		185 (35.1 %)		189 (35.7 %)		182 (34.3 %)		769 (34.6 %)
1–3 times in the last year	25 (23.1 %)		98 (18.6 %)		94 (17.8 %)		100 (18.9 %)		106 (20.0 %)		423 (19.1 %)
4–11 times in the last year	17 (15.7 %)		98 (18.6 %)		92 (17.5 %)		89 (16.8 %)		95 (17.9 %)		391 (17.6 %)
1–3 times per month	18 (16.7 %)		82 (15.6 %)		80 (15.2 %)		66 (12.5 %)		79 (14.9 %)		325 (14.6 %)
Once per week or more often	14 (13.0 %)		67 (12.7 %)		75 (14.2 %)		85 (16.1 %)		62 (11.7 %)		303 (13.6 %)
Prefer not to say	0 (0 %)		2 (0.4 %)		1 (0.2 %)		0 (0 %)		6 (1.1 %)		9 (0.4 %)
**Importance of food sustainability**											
Very important	6 (5.6 %)		53 (10.1 %)		55 (10.4 %)		46 (8.7 %)		45 (8.5 %)		205 (9.2 %)
Important	20 (18.5 %)		129 (24.5 %)		126 (23.9 %)		117 (22.1 %)		135 (25.5 %)		527 (23.7 %)
Moderately important	47 (43.5 %)		164 (31.2 %)		177 (33.6 %)		157 (29.7 %)		162 (30.6 %)		707 (31.8 %)
Slightly important	17 (15.7 %)		98 (18.6 %)		80 (15.2 %)		108 (20.4 %)		102 (19.2 %)		405 (18.2 %)
Not important	18 (16.7 %)		81 (15.4 %)		89 (16.9 %)		101 (19.1 %)		86 (16.2 %)		375 (16.9 %)
Missing	0 (0 %)		1 (0.2 %)		0 (0 %)		0 (0 %)		0 (0 %)		1 (0.0 %)
**Importance of food healthiness**											
Very important	22 (20.4 %)		119 (22.6 %)		131 (24.9 %)		121 (22.9 %)		99 (18.7 %)		492 (22.2 %)
Important	38 (35.2 %)		192 (36.5 %)		187 (35.5 %)		186 (35.2 %)		200 (37.7 %)		803 (36.2 %)
Moderately important	31 (28.7 %)		152 (28.9 %)		156 (29.6 %)		155 (29.3 %)		166 (31.3 %)		660 (29.7 %)
Slightly important	13 (12.0 %)		42 (8.0 %)		42 (8.0 %)		41 (7.8 %)		44 (8.3 %)		182 (8.2 %)
Not important	4 (3.7 %)		20 (3.8 %)		10 (1.9 %)		25 (4.7 %)		20 (3.8 %)		79 (3.6 %)
Missing	0 (0 %)		1 (0.2 %)		1 (0.2 %)		1 (0.2 %)		1 (0.2 %)		4 (0.2 %)
**Weekly shopping expenses**											
Mean (SD)	104.6 (89.7)		95.9 (74.6)		102.4 (103.4)		93.7 (77.0)		89.9 (73.3)		95.9 (83.3)
Median [Min, Max]	95.0 [2.00, 800]		80.0 [5.00, 1000]		80.0 [10.0, 1000]		80.0 [1.00, 1000]		75.0 [10.0, 1000]		80.0 [1.00, 1000]
Missing	3 (2.8 %)		12 (2.3 %)		22 (4.2 %)		19 (3.6 %)		14 (2.6 %)		70 (3.2 %)
**Household size**											
Mean (SD)	2.59 (1.21)		2.58 (1.31)		2.55 (1.30)		2.54 (1.27)		2.50 (1.30)		2.54 (1.29)
Median [Min, Max]	2.00 [1.00, 6.00]		2.00 [1.00, 8.00]		2.00 [1.00, 9.00]		2.00 [1.00, 8.00]		2.00 [1.00, 10.0]		2.00 [1.00, 10.0]
Missing	1 (0.9 %)		4 (0.8 %)		2 (0.4 %)		5 (0.9 %)		3 (0.6 %)		15 (0.7 %)

*Note*. GCSE: General Certificate of Secondary Education, UK qualifications usually sat at around age 16; A-levels: Advanced-level qualifications, usually taken around age 18. Education categories based on UK census categories: None; Up to 4 GCSE’s (Including 1–4 O Levels/CSE/GCSEs (any grades), Foundation Diploma, NVQ level 1, Foundation GNVQ or equivalents); 5 or more GCSE’s or 1 A-level (Including 5+ GCSEs (Grades A*-C),1 A Level/2–3 AS Levels, NVQ level 2, Intermediate GNVQ, City and Guilds Craft, BTEC First/General Diploma, RSA Diploma, Apprenticeship or equivalents); 2 or more A-levels (Including 2+ A Levels, 4+ AS Levels, NVQ Level 3, Advanced GNVQ, City and Guilds Advanced Craft, ONC, OND, BTEC National, RSA Advanced Diploma or equivalents); Bachelor’s degree (Including BA, BSc, NVQ Level 4–5, HNC, HND, RSA Higher Diploma, BTEC Higher level or equivalents) or Post-Graduate degree or qualification (Including Higher Degrees e.g. MA, PhD, PGCE, Professional qualifications e.g. teaching, nursing, accountancy or equivalents). Category “None” combined in analysis with “Up to 4 GCSE’s”. Meat consumption: Participants were asked in three separate questions how often per week they consume meat for a. breakfast, b. lunch, c. dinner. Answers to each of the three questions received a score: 0 for “Less than once a week”, 1 for “1–2 days a week”, 2 for “3–4 days a week”, 3 for “5–6 days a week”, 4 for “Every day”. Scores were then summed to obtain an overall meat consumption score, categorised as low (score between 0 and 4), medium (score between 5 and 8) or high (score between 9 and 12).

**Table 3:** Mean and Standard Deviations for Nutri-Score and eco score in each study condition.

	Mean (SD) Nutri-Score	Mean (SD) Eco Score
*Control*	35.71 (3.53)	4.67 (2.17)
*Health Position*	33.41 (3.57)	4.52 (2.04)
*Health Position & Labels*	33.21 (3.96)	4.64 (2.12)
*Eco Position*	37.19 (3.98)	3.68 (2.05)
*Eco Position & Labels*	36.66 (3.74)	3.40 (1.96)

**Table 4:** Regression analyses of mean (i) Nutri-Score or (ii) Eco score of shopping baskets.

Nutri-Score		N		Reference group		Comparison		B^a^		95 % CI low		95 % CI high		p-value
*Primary analyses*		*1161*		*Control*		*Health Position*		−2.30		−3.07		−1.52		<0.001 (*)
						*Health Position & Labels*		–2.50		−3.28		–1.72		<0.001 (*)
		*1053*		*Health Position*		*Health Position & Labels*		–0.20		–0.66		0.25		0.380
*Sensitivity analyses (full compliance)*		*583*		*Control*		*Health Position*		–2.53		– 3.62		–1.44		<0.001 (*)
						*Health Position & Labels*		–3.17		– 4.27		– 2.08		<0.001 (*)
		*537*		*Health Position*		*Health Position & Labels*		–0.64		–1.23		–0.05		0.033
**Eco score**		**N**		**Reference group**		**Comparison**		**Exp(B) ** ^a^		**95 % CI low**		**95 % CI high**		**p-value**
*Primary analyses*		*1167*		*Control*		*Eco Position*		0.76		0.69		0.85		<0.001 (*)
						*Eco Position & Labels*		0.70		0.63		0.78		<0.001 (*)
		*1059*		*Eco Position*		*Eco Position & Labels*		0.92		0.86		0.98		0.008 (*)
*Sensitivity analyses (full compliance)*		*561*		*Control*		*Eco Position*		0.77		0.65		0.91		0.002 (*)
						*Eco Position & Labels*		0.71		0.60		0.84		<0.001 (*)
		*515*		*Eco Position*		*Eco Position & Labels*		0.92		0.84		1.01		0.069

a B (non-exponentiated coefficients) are presented for Nutri-Score analyses; Exponentiated coefficients are presented for eco score analyses;

(*) denotes statistical significance at a threshold of p < 0.025.

**Table 5:** Impact of health position and health position & labels interventions vs. control on mean a) energy density (b) salt, c) fat, d) sugar, e) price (£GBP) and f) eco score of products in shopping baskets.

		Estimate (B)^a^	95 % Conf. low	95 % Conf. high	p-value
*Energy density*	*Health Position*	−5.73	–10.34	–1.12	0.015
	*Health Position & Labels*	–7.08	–11.69	–2.47	0.003 ^b^
*Salt*	*Health Position*	–0.04	–0.06	–0.01	0.004 ^b^
	*Health Position & Labels*	–0.06	–0.09	–0.04	<0.001 (*)
*Fat*	*Health Position*	–0.67	–1.10	–0.24	0.002 ^b^
	*Health Position & Labels*	–0.78	–1.21	–0.35	<0.001 (*)
*Sugar*	*Health Position*	0.02	–0.24	0.28	0.871
	*Health Position & Labels*	–0.06	–0.31	0.20	0.669
*Price*	*Health Position*	–1.31	–2.62	0.01	0.051
	*Health Position & Labels*	–1.64	–2.95	–0.32	0.010 ^b^
*Eco score*	*Health Position*	Exp(B) = 0.98	0.90	1.07	0.696
	*Health Position & Labels*	Exp(B) = 1.01	0.92	1.10	0.892

a Unless otherwise stated; Exponentiated coefficients presented for eco score analyses.

b marks significance compared to our threshold of p < 0.005 for nutrient analyses. For price, a Tukey test was performed, and a significance threshold of p < 0.025 was used. The eco-score outcome was exploratory, so p values are presented but no pre-specified significance threshold was set, as we will not draw conclusions from these results.

**Table 6:** Impact of eco position and eco position & labels interventions vs. control on log-transformed mean a) greenhouse gas emissions (GHG), b) biodiversity loss, c) water scarcity, d) eutrophication, e) price and f) Nutri-Score of products in shopping baskets.

		Estimate (Exp(B))^a^	95 % Conf. low	95 % Conf. high	p-value
*GHGs*	*Eco Position*	0.76	0.68	0.85	<0.001 ^b^
	*Eco Position*	0.69	0.61	0.77	<0.001
	*& Labels*				^b^
*Biodiversity*	*Eco Position*	0.79	0.70	0.89	<0.001 ^b^
	*Eco Position*	0.72	0.64	0.81	<0.001
	*& Labels*				^b^
*Water use*	*Eco Position*	0.75	0.69	0.81	<0.001 ^b^
	*Eco Position*	0.71	0.66	0.76	<0.001
	*& Labels*				^b^
*Eutrophication*	*Eco Position*	0.78	0.67	0.91	0.002 ^b^
	*Eco Position*	0.70	0.60	0.81	<0.001
	*& Labels*				^b^
*Price*	*Eco Position*	B = –1.61	–2.81	–0.40	0.010 ^b^
	*Eco Position*	B = –1.75	– 2.96	–0.55	0.002 ^b^
	*& Labels*				
*Nutri-Score*	*Eco Position*	B = 1.48	0.68	2.27	<0.001
	*Eco Position*	B = 0.95	0.16	1.75	0.019
	*& Labels*				

a Coefficients and confidence intervals were exponentiated to facilitate interpretation unless otherwise stated; non-exponentiated B coefficients presented for price and nutri-score analyses.

b marks significance compared to our threshold of p < 0.005 for environmental indicator analyses. For price, a Welch ANOVA and Games-Howell post-hoc test was performed due to a significant Levene’s test (p = 0.04), and a significance threshold of p < 0.025 was used. The nutri-score outcome was exploratory, so p values are presented but no pre-specified significance threshold was set, as we will not draw conclusions from these results.

## Data Availability

Data are available on OSF (https://osf.io/tcwr5/files/258ca).
