# Supplementary material for: Combining positioning and labelling interventions for healthier and more environmentally sustainable products: A randomised controlled trial in an online experimental supermarket
Source: Appetite. Author manuscript; Available in PMC 2026 Feb 19. (PMC7618743; doi:10.1016/j.appet.2025.108378)
Supplement: Supplementary information [file EMS211094-supplement-Supplementary_information.docx]

**Appendix**

**Table of Contents**

[Appendix A: Qualtrics survey questions 2](#_Toc207360298)

[Appendix B: Coding of participant characteristics for linear regressions 6](#_Toc207360299)

[Appendix C: Acceptability and shopping habit questions in the post-intervention survey 6](#_Toc207360300)

[Appendix D. Analysis without participants who bought affected products (in sustainability & labels group) and with affected products removed 8](#_Toc207360301)

[Appendix E. Energy and salt models without outliers 8](#_Toc207360302)

[Appendix F. Health interaction models 9](#_Toc207360303)

[Appendix G. Sustainability interaction models 11](#_Toc207360304)

[Appendix H. Interaction models for a) health and b) environmental consciousness 13](#_Toc207360305)

[Appendix I. Adjusted models comparing positioning & label groups to positioning only 13](#_Toc207360306)

[Appendix J. Secondary outcomes by study condition 15](#_Toc207360307)

[Appendix K. Tukey test of price for sustainability intervention groups and control, with outliers removed 16](#_Toc207360308)

[Appendix L. Impact of eco position and eco position & labels interventions on mean Nutri-Score of products in shopping baskets, model with outliers removed 16](#_Toc207360309)

# Appendix A: Qualtrics survey questions

Screening Survey:

From all participants completing the study, ten will be randomly selected to win an item of a value of up to £5 selected at random in their shopping basket. Participation is optional.

- Yes, I want to opt-in for a chance to win one randomly selected item in my shopping basket
- No, I do not want to opt-in

Are you:

- Under 18 years old?
- 18 years old or over?

Do you currently reside in the UK?

- Yes
- No

Are you fluent in English?

- Yes
- No

Baseline Survey:

1. Demographic characteristics

- Please indicate what gender you identify with
  - Male
  - Female
  - Other gender identity
- Please tell us your age (in years)
  - [FREE TEXT ANSWER]

1. Please indicate your household income (total household income before tax)

- Below £15.5K
- Between £15.5K up to and including £25K
- Between £25K and £39K
- £40K or above
- Prefer not to say

1. Household size – “How many people live at your house, including you?
   - Please specify (in numbers)
     - [FREE TEXT ANSWER]
   - Prefer not to say
2. Highest level of qualification - “What is the highest education qualification you have achieved?” (categories based on UK 2021 census categories)

- No qualifications
- Level 1 and entry level qualifications: 1 to 4 GCSEs grade A* to C, Any GCSEs at other grades, O levels or CSEs (any grades), 1 AS level, NVQ level 1, Foundation GNVQ, Basic or Essential Skills
- Level 2 qualifications: 5 or more GCSEs (A* to C or 9 to 4), O levels (passes), CSEs (grade 1), School Certification, 1 A level, 2 to 3 AS levels, VCEs, Intermediate or Higher Diploma, Welsh Baccalaureate Intermediate Diploma, NVQ level 2, Intermediate GNVQ, City and Guilds Craft, BTEC First or General Diploma, RSA Diploma
- Level 3 qualifications: 2 or more A levels or VCEs, 4 or more AS levels, Higher School Certificate, Progression or Advanced Diploma, Welsh Baccalaureate Advance Diploma, NVQ level 3; Advanced GNVQ, City and Guilds Advanced Craft, ONC, OND, BTEC National, RSA Advanced Diploma
- Level 4 qualifications or above: degree (BA, BSc), higher degree (MA, PhD, PGCE), NVQ level 4 to 5, HNC, HND, RSA Higher Diploma, BTEC Higher level, professional qualifications (for example, teaching, nursing, accountancy)

1. Regular shopping

“On average, how much do you spend on supermarket shopping per week?” (answer in £)

- - **Please specify** (in numbers)
    - [FREE TEXT ANSWER]
  - Prefer not to say

1. Online shopping experience

“How often, on average over the past year, have you shopped online for food or groceries to be delivered to you (e.g. Tesco.com, Ocado.com, mysupermarket.co.uk)?”

- Never or not in the last year
- 1-3 times in the last year
- 4-11 times in the last year
- 1-3 per month
- Once per week or more often.
- Prefer not to say

1. Do you have any dietary restrictions?

- Vegan
- Vegetarian
- Gluten-free
- Sugar-free
- Dairy/lactose-free
- None

1. How many times a week do you typically eat meat at breakfast/lunch/dinner?

|  | Less than once a week | 1-2 days a week | 3-4 days a week | 5-6 days a week | Every day | Prefer not to say |
| --- | --- | --- | --- | --- | --- | --- |
| Breakfast |  |  |  |  |  |  |
| Lunch |  |  |  |  |  |  |
| Dinner |  |  |  |  |  |  |

1. How many times a week do you typically eat dairy (e.g. butter, milk, cheese) at breakfast/lunch/dinner?

|  | Less than once a week | 1-2 days a week | 3-4 days a week | 5-6 days a week | Every day | Prefer not to say |
| --- | --- | --- | --- | --- | --- | --- |
| Breakfast |  |  |  |  |  |  |
| Lunch |  |  |  |  |  |  |
| Dinner |  |  |  |  |  |  |

Post-Intervention Survey:

1. Do you often think about the environmental impact of the foods you select when doing your shopping?

- Strongly agree
- Somewhat agree
- Indifferent
- Somewhat disagree
- Strongly disagree

1. Do you often think about the healthiness of the foods you select when doing your shopping?

- Strongly agree
- Somewhat agree
- Indifferent
- Somewhat disagree
- Strongly disagree

1. If supermarkets were to introduce a feature that positioned products to emphasise products with a lower-environmental impact, to what extent would you support or oppose this?

- Strongly support
- Support
- Somewhat support
- Neither support nor oppose
- Somewhat oppose
- Oppose
- Strongly oppose

1. If supermarkets were to introduce a feature that positioned products to emphasise healthier products, to what extent would you support or oppose this?

- Strongly support
- Support
- Somewhat support
- Neither support nor oppose
- Somewhat oppose
- Oppose
- Strongly oppose

1. If supermarkets were to label products to indicate their environmental impact, to what extent would you support or oppose this?

- Strongly support
- Support
- Somewhat support
- Neither support nor oppose
- Somewhat oppose
- Oppose
- Strongly oppose

1. Do you often use the front-of-pack nutrition labelling to decide which foods to select when doing your shopping?

- Strongly agree
- Somewhat agree
- Indifferent
- Somewhat disagree
- Strongly disagree

1. “Is there anything else you’d like to tell us about your experience with this shopping task today?” (Please do NOT include your name here).

[FREE TEXT ANSWER]

# Appendix B: Coding of participant characteristics for linear regressions

| **Original measures** | **Aggregated group for models** |
| --- | --- |
| **Gender identity** | |
| *Female* | Female |
| *Male* | Male |
| *Other gender identity* | Excluded from models due to very small group size |
| **Age group** | |
| *18-24* | 18-34 |
| *25-34* |  |
| *35-44* | 35-54 |
| *45-54* |  |
| *55-64* | 55+ |
| *65+* |  |
| **Education** | |
| *No qualifications* | Lower |
| *Level 1 and entry level* |  |
| *Level 2* |  |
| *Level 3* | Higher |
| *Level 4* |  |
| **Household income** | |
| *Below £15.5K* | £0-25K |
| *Between £15.5K up to and including £25K* |  |
| *Between £25K and £39K* | £25K and above |
| *£40K or above* |  |
| *Prefer not to say* | Prefer not to say (excluded from income interaction models due to small group size) |
| **Meat consumption** | |
| *Low* | Low |
| *Medium* | Medium |
| *High* | High |
| **Importance of food healthiness** | |
| *Very important* | Very important or important |
| *Important* |  |
| *Moderately important* | Moderately important |
| *Slightly important* | Not or slightly important |
| *Not important* |  |
| **Importance of food sustainability** |  |
| *Very important* | Very important or important |
| *Important* |  |
| *Moderately important* | Moderately important |
| *Slightly important* | Not or slightly important |
| *Not important* |  |

#

# Appendix C: Acceptability and shopping habit questions in the post-intervention survey

1. How important to you is the environmental impact of the foods you select when doing your shopping?

- Very important
- Important
- Moderately important
- Slightly important
- Not important

1. How important to you is the healthiness of the foods you select when doing your shopping?

- Very important
- Important
- Moderately important
- Slightly important
- Not important

1. If supermarkets were to position products to emphasise more environmentally sustainable products, to what extent would you support or oppose this?

- Strongly support
- Support
- Somewhat support
- Neither support nor oppose
- Somewhat oppose
- Oppose
- Strongly oppose

1. If supermarkets were to position products to emphasise healthier products, to what extent would you support or oppose this?

- Strongly support
- Support
- Somewhat support
- Neither support nor oppose
- Somewhat oppose
- Oppose
- Strongly oppose

1. If supermarkets were to label products to indicate their environmental impact, to what extent would you support or oppose this?

- Strongly support
- Support
- Somewhat support
- Neither support nor oppose
- Somewhat oppose
- Oppose
- Strongly oppose

1. How frequently do you use the front-of-pack nutrition labelling to decide which foods to select when doing your shopping?

- Always
- Often
- Sometimes
- Rarely
- Never

# Appendix D. Analysis without participants who bought affected products (in sustainability & labels group) and with affected products removed

|  | **Estimate** | **Std. error** | **Statistic** | **p-value** | **95% Conf. low** | **95% Conf. high** |
| --- | --- | --- | --- | --- | --- | --- |
| 1. **Health groups vs. control** | | | | | | |
| *Intercept* | 34.97 | 0.32 | 108.42 | 0.000 | 34.34 | 35.61 |
| *Health Position* | -2.36 | 0.35 | -6.66 | 0.000 (*) | -3.05 | -1.66 |
| *Health Position & Labels* | -2.57 | 0.36 | -7.15 | 0.000 (*) | -3.28 | -1.87 |
| 1. **Sensitivity analysis health groups vs. control** | | | | | | |
| *Intercept* | 35.63 | 0.47 | 76.06 | 0.000 | 34.71 | 36.55 |
| *Health Position* | -2.74 | 0.51 | -5.42 | 0.000 (*) | -3.74 | -1.75 |
| *Health Position & Labels* | -3.23 | 0.51 | -6.28 | 0.000 (*) | -4.24 | -2.22 |
| 1. **Health position & labels vs. health position** | | | | | | |
| *Intercept* | 32.61 | 0.15 | 223.59 | 0.000 | 32.33 | 32.90 |
| *Health Position & Labels* | -0.22 | 0.22 | -1.00 | 0.319 | -0.64 | 0.21 |
| 1. **Sensitivity analysis health position & labels vs health position** | | | | | | |
| *Intercept* | 32.89 | 0.19 | 172.06 | 0.000 | 32.52 | 33.27 |
| *Health Position & Labels* | -0.49 | 0.29 | -1.71 | 0.088 | -1.05 | 0.07 |

*Note.* (*) labels significance compared to our threshold of p<0.025

# Appendix E. Energy and salt models without outliers

|  | **Estimate** | **Std. error** | **Statistic** | **p-value** | **95% Conf. low** | **95% Conf. high** |
| --- | --- | --- | --- | --- | --- | --- |
| 1. **Energy** | | | | | | |
| *Control (ref)* | 220.83 | 1.95 | 113.30 | 0.000 | 217.00 | 224.65 |
| *Health Position* | -6.88 | 2.14 | -3.22 | 0.001 (*) | -11.07 | -2.68 |
| *Health Position & Labels* | -7.86 | 2.14 | -3.68 | 0.000 (*) | -12.06 | -3.66 |
| 1. **Salt** |  |  |  |  |  |  |
| *Control (ref)* | 0.75 | 0.01 | 68.98 | 0.000 | 0.73 | 0.77 |
| *Health Position* | -0.02 | 0.01 | -1.38 | 0.169 | -0.04 | 0.01 |
| *Health Position & Labels* | -0.04 | 0.01 | -3.62 | 0.000 (*) | -0.07 | -0.02 |

*Note.* (*) labels significance compared to our threshold of p<0.005

# Appendix F. Health interaction models

|  | **Estimate** | **Standard error** | **Statistic** | **p-value** | **95% conf. interval low** | **95% conf. interval high** |
| --- | --- | --- | --- | --- | --- | --- |
| **Gender interaction model** | | | | | | |
| *Intercept* | 35.81 | 0.56 | 64.22 | 0.000 | 34.72 | 36.91 |
| *Group: Health Position* | -2.16 | 0.52 | -4.13 | 0.000 | -3.19 | -1.14 |
| *Group: Health Position & Labels* | -2.30 | 0.53 | -4.36 | 0.000 | -3.34 | -1.27 |
| *Gender: Male* | 0.40 | 0.73 | 0.55 | 0.584 | -1.03 | 1.83 |
| *Age: 18-24* | -0.71 | 0.29 | -2.45 | 0.015 | -1.28 | -0.14 |
| *Age: 35-54* | -0.12 | 0.26 | -0.47 | 0.638 | -0.64 | 0.39 |
| *Income: £0-25K* | 0.75 | 0.25 | 3.00 | 0.003 | 0.26 | 1.24 |
| *Income: Prefer not to say* | 1.24 | 0.55 | 2.27 | 0.023 | 0.17 | 2.31 |
| *Education: Lower* | 0.02 | 0.23 | 0.08 | 0.936 | -0.43 | 0.47 |
| *Meat consumption: Medium* | -0.43 | 0.27 | -1.56 | 0.118 | -0.96 | 0.11 |
| *Meat consumption: Low* | -0.57 | 0.31 | -1.81 | 0.070 | -1.18 | 0.05 |
| *Group: Health Position * Gender: Male* | -0.29 | 0.80 | -0.37 | 0.715 | -1.86 | 1.27 |
| *Group: Health Position & Labels * Gender: Male* | -0.44 | 0.80 | -0.55 | 0.583 | -2.00 | 1.13 |
| **Age group interaction model** | | | | | | |
| *Intercept* | 35.84 | 0.62 | 58.25 | 0.000 | 34.63 | 37.05 |
| *Group: Health Position* | -2.04 | 0.61 | -3.36 | 0.001 | -3.23 | -0.85 |
| *Group: Health Position & Labels* | -2.51 | 0.61 | -4.12 | 0.000 | -3.71 | -1.32 |
| *Gender: Male* | 0.08 | 0.23 | 0.37 | 0.711 | -0.36 | 0.53 |
| *Age: 18-24* | -0.19 | 0.93 | -0.20 | 0.839 | -2.02 | 1.64 |
| *Age: 35-54* | -0.16 | 0.83 | -0.19 | 0.846 | -1.79 | 1.47 |
| *Income: £0-25K* | 0.73 | 0.25 | 2.94 | 0.003 | 0.24 | 1.22 |
| *Income: Prefer not to say* | 1.22 | 0.55 | 2.23 | 0.026 | 0.15 | 2.29 |
| *Education: Lower* | 0.03 | 0.23 | 0.13 | 0.899 | -0.42 | 0.48 |
| *Meat consumption: Medium* | -0.43 | 0.27 | -1.56 | 0.120 | -0.96 | 0.11 |
| *Meat consumption: Low* | -0.56 | 0.31 | -1.79 | 0.073 | -1.18 | 0.05 |
| *Group: Health Position * Age: 18-34* | -1.00 | 1.01 | -0.98 | 0.325 | -2.99 | 0.99 |
| *Group: Health Position & Labels * Age: 18-34* | -0.13 | 1.01 | -0.13 | 0.895 | -2.12 | 1.86 |
| *Group: Health Position * Age: 35-54* | -0.01 | 0.91 | -0.01 | 0.990 | -1.80 | 1.77 |
| *Group: Health Position & Labels * Age: 35-54* | 0.09 | 0.91 | 0.10 | 0.917 | -1.69 | 1.88 |
| **Income interaction model** | | | | | | |
| *Intercept* | 35.97 | 0.53 | 67.24 | 0.000 | 34.92 | 37.02 |
| *Group: Health Position* | -2.16 | 0.49 | -4.41 | 0.000 | -3.12 | -1.20 |
| *Group: Health Position & Labels* | -2.71 | 0.49 | -5.53 | 0.000 | -3.68 | -1.75 |
| *Gender: Male* | 0.08 | 0.23 | 0.36 | 0.715 | -0.37 | 0.53 |
| *Age: 18-24* | -0.72 | 0.30 | -2.43 | 0.015 | -1.31 | -0.14 |
| *Age: 35-54* | -0.05 | 0.27 | -0.20 | 0.844 | -0.58 | 0.47 |
| *Income: £0-25K* | 0.69 | 0.79 | 0.87 | 0.382 | -0.86 | 2.24 |
| *Education: Lower* | 0.02 | 0.24 | 0.10 | 0.920 | -0.44 | 0.49 |
| *Meat consumption: Medium* | -0.46 | 0.28 | -1.64 | 0.101 | -1.01 | 0.09 |
| *Meat consumption: Low* | -0.53 | 0.32 | -1.64 | 0.101 | -1.17 | 0.10 |
| *Group: Health Position * Income: £0-25K* | -0.19 | 0.86 | -0.22 | 0.829 | -1.88 | 1.51 |
| *Group: Health Position & Labels * Income: £0-25K* | 0.33 | 0.86 | 0.38 | 0.702 | -1.36 | 2.02 |
| **Education interaction model** | | | | | | |
| *Intercept* | 36.18 | 0.54 | 66.88 | 0.000 | 35.12 | 37.24 |
| *Group: Health Position* | -2.56 | 0.51 | -5.05 | 0.000 | -3.56 | -1.57 |
| *Group: Health Position & Labels* | -2.72 | 0.51 | -5.32 | 0.000 | -3.73 | -1.72 |
| *Gender: Male* | 0.07 | 0.23 | 0.30 | 0.761 | -0.37 | 0.51 |
| *Age: 18-24* | -0.70 | 0.29 | -2.42 | 0.016 | -1.27 | -0.13 |
| *Age: 35-54* | -0.12 | 0.26 | -0.46 | 0.643 | -0.64 | 0.39 |
| *Income: £0-25K* | 0.74 | 0.25 | 2.96 | 0.003 | 0.25 | 1.23 |
| *Income: Prefer not to say* | 1.24 | 0.55 | 2.27 | 0.024 | 0.17 | 2.31 |
| *Education: Lower* | -0.55 | 0.74 | -0.75 | 0.453 | -2.00 | 0.89 |
| *Meat consumption: Medium* | -0.43 | 0.27 | -1.58 | 0.114 | -0.97 | 0.10 |
| *Meat consumption: Low* | -0.57 | 0.31 | -1.82 | 0.069 | -1.19 | 0.04 |
| *Group: Health Position * Education: Lower* | 0.70 | 0.81 | 0.87 | 0.387 | -0.89 | 2.28 |
| *Group: Health Position & Labels * Education: Lower* | 0.57 | 0.81 | 0.71 | 0.478 | -1.01 | 2.16 |
| **Meat consumption interaction model** | | | | | | |
| *Intercept* | 35.84 | 0.79 | 45.60 | 0.000 | 34.30 | 37.38 |
| *Group: Health Position* | -2.31 | 0.82 | -2.82 | 0.005 | -3.91 | -0.70 |
| *Group: Health Position & Labels* | -2.27 | 0.81 | -2.81 | 0.005 | -3.86 | -0.69 |
| *Gender: Male* | 0.08 | 0.23 | 0.36 | 0.720 | -0.36 | 0.52 |
| *Age: 18-24* | -0.70 | 0.29 | -2.42 | 0.015 | -1.27 | -0.13 |
| *Age: 35-54* | -0.12 | 0.26 | -0.46 | 0.648 | -0.64 | 0.40 |
| *Income: £0-25K* | 0.74 | 0.25 | 2.96 | 0.003 | 0.25 | 1.23 |
| *Income: Prefer not to say* | 1.24 | 0.55 | 2.27 | 0.023 | 0.17 | 2.32 |
| *Education: Lower* | 0.04 | 0.23 | 0.15 | 0.877 | -0.42 | 0.49 |
| *Meat consumption: Medium* | -0.23 | 0.90 | -0.26 | 0.795 | -2.00 | 1.53 |
| *Meat consumption: Low* | -0.56 | 1.04 | -0.53 | 0.593 | -2.60 | 1.49 |
| *Group: Health Position * Meat consumption: Medium* | 0.06 | 0.99 | 0.06 | 0.951 | -1.88 | 2.00 |
| *Group: Health Position & Labels * Meat consumption: Medium* | -0.48 | 0.98 | -0.49 | 0.625 | -2.40 | 1.44 |
| *Group: Health Position * Meat consumption: Low* | 0.00 | 1.13 | 0.00 | 0.997 | -2.22 | 2.21 |
| *Group: Health Position & Labels * Meat consumption: Low* | 0.02 | 1.13 | 0.02 | 0.983 | -2.19 | 2.24 |

# Appendix G. Sustainability interaction models

|  | **Estimate** | **Standard error** | **Statistic** | **p-value** | **95% conf. interval low** | **95% conf. interval high** |
| --- | --- | --- | --- | --- | --- | --- |
| **Gender interaction model** | | | | | | |
| *Intercept* | 3.65 | 0.07 | 17.40 | 0.000 | 3.15 | 4.22 |
| *Group: Eco Position* | 0.84 | 0.07 | -2.44 | 0.015 | 0.73 | 0.97 |
| *Group: Eco Position & Labels* | 0.81 | 0.07 | -3.00 | 0.003 | 0.70 | 0.93 |
| *Gender: Male* | 1.22 | 0.10 | 2.00 | 0.045 | 1.00 | 1.47 |
| *Age: 18-24* | 0.84 | 0.04 | -4.58 | 0.000 | 0.78 | 0.91 |
| *Age: 35-54* | 1.01 | 0.04 | 0.28 | 0.782 | 0.94 | 1.08 |
| *Income: £0-25K* | 1.06 | 0.03 | 1.86 | 0.064 | 1.00 | 1.14 |
| *Income: Prefer not to say* | 0.98 | 0.07 | -0.23 | 0.817 | 0.85 | 1.14 |
| *Education: Lower* | 1.10 | 0.03 | 3.19 | 0.001 | 1.04 | 1.17 |
| *Meat consumption: Medium* | 1.11 | 0.04 | 3.02 | 0.003 | 1.04 | 1.19 |
| *Meat consumption: Low* | 0.94 | 0.04 | -1.64 | 0.101 | 0.86 | 1.01 |
| *Group: Eco Position * Gender: Male* | 0.82 | 0.11 | -1.84 | 0.067 | 0.67 | 1.01 |
| *Group: Eco Position & Labels * Gender: Male* | 0.76 | 0.11 | -2.62 | 0.009 | 0.61 | 0.93 |
| **Age group interaction model** | | | | | | |
| *Intercept* | 4.32 | 0.08 | 17.71 | 0.000 | 3.67 | 5.08 |
| *Group: Eco Position* | 0.69 | 0.08 | -4.55 | 0.000 | 0.59 | 0.81 |
| *Group: Eco Position & Labels* | 0.69 | 0.08 | -4.50 | 0.000 | 0.59 | 0.81 |
| *Gender: Male* | 0.98 | 0.03 | -0.77 | 0.441 | 0.92 | 1.04 |
| *Age: 18-24* | 0.69 | 0.13 | -2.95 | 0.003 | 0.54 | 0.88 |
| *Age: 35-54* | 0.94 | 0.11 | -0.59 | 0.552 | 0.75 | 1.16 |
| *Income: £0-25K* | 1.06 | 0.03 | 1.88 | 0.061 | 1.00 | 1.14 |
| *Income: Prefer not to say* | 0.98 | 0.07 | -0.25 | 0.801 | 0.85 | 1.14 |
| *Education: Lower* | 1.11 | 0.03 | 3.27 | 0.001 | 1.04 | 1.18 |
| *Meat consumption: Medium* | 1.10 | 0.04 | 2.82 | 0.005 | 1.03 | 1.18 |
| *Meat consumption: Low* | 0.93 | 0.04 | -1.85 | 0.065 | 0.86 | 1.00 |
| *Group: Eco Position * Age: 18-34* | 1.39 | 0.14 | 2.43 | 0.015 | 1.06 | 1.81 |
| *Group: Eco Position & Labels * Age: 18-34* | 1.09 | 0.14 | 0.64 | 0.519 | 0.84 | 1.43 |
| *Group: Eco Position * Age: 35-54* | 1.10 | 0.12 | 0.79 | 0.428 | 0.87 | 1.40 |
| *Group: Eco Position & Labels * Age: 35-54* | 1.05 | 0.12 | 0.43 | 0.667 | 0.83 | 1.34 |
| **Income interaction model** | | | | | | |
| *Intercept* | 3.99 | 0.07 | 19.29 | 0.000 | 3.47 | 4.59 |
| *Group: Eco Position* | 0.79 | 0.07 | -3.52 | 0.000 | 0.70 | 0.90 |
| *Group: Eco Position & Labels* | 0.73 | 0.07 | -4.75 | 0.000 | 0.64 | 0.83 |
| *Gender: Male* | 0.96 | 0.03 | -1.23 | 0.220 | 0.91 | 1.02 |
| *Age: 18-24* | 0.82 | 0.04 | -4.97 | 0.000 | 0.76 | 0.89 |
| *Age: 35-54* | 1.02 | 0.04 | 0.41 | 0.679 | 0.94 | 1.09 |
| *Income: £0-25K* | 1.11 | 0.11 | 0.98 | 0.329 | 0.90 | 1.37 |
| *Education: Lower* | 1.10 | 0.03 | 3.08 | 0.002 | 1.04 | 1.17 |
| *Meat consumption: Medium* | 1.10 | 0.04 | 2.73 | 0.007 | 1.03 | 1.18 |
| *Meat consumption: Low* | 0.93 | 0.04 | -1.72 | 0.086 | 0.86 | 1.01 |
| *Group: Eco Position * Income: £0-25K* | 0.96 | 0.12 | -0.36 | 0.720 | 0.76 | 1.21 |
| *Group: Eco Position & Labels * Income: £0-25K* | 0.95 | 0.12 | -0.42 | 0.677 | 0.76 | 1.20 |
| **Education interaction model** | | | | | | |
| *Intercept* | 3.89 | 0.07 | 18.76 | 0.000 | 3.38 | 4.49 |
| *Group: Eco Position* | 0.81 | 0.07 | -3.09 | 0.002 | 0.71 | 0.93 |
| *Group: Eco Position & Labels* | 0.73 | 0.07 | -4.63 | 0.000 | 0.64 | 0.83 |
| *Gender: Male* | 0.98 | 0.03 | -0.72 | 0.470 | 0.92 | 1.04 |
| *Age: 18-24* | 0.84 | 0.04 | -4.67 | 0.000 | 0.78 | 0.90 |
| *Age: 35-54* | 1.00 | 0.04 | 0.12 | 0.904 | 0.94 | 1.08 |
| *Income: £0-25K* | 1.07 | 0.03 | 1.94 | 0.053 | 1.00 | 1.14 |
| *Income: Prefer not to say* | 0.98 | 0.07 | -0.23 | 0.814 | 0.85 | 1.14 |
| *Education: Lower* | 1.19 | 0.10 | 1.71 | 0.088 | 0.98 | 1.44 |
| *Meat consumption: Medium* | 1.11 | 0.04 | 3.08 | 0.002 | 1.04 | 1.19 |
| *Meat consumption: Low* | 0.94 | 0.04 | -1.59 | 0.111 | 0.87 | 1.02 |
| *Group: Eco Position * Education: Lower* | 0.90 | 0.11 | -0.99 | 0.323 | 0.72 | 1.11 |
| *Group: Eco Position & Labels * Education: Lower* | 0.95 | 0.11 | -0.44 | 0.657 | 0.77 | 1.18 |
| **Meat consumption interaction model** | | | | | | |
| *Intercept* | 4.10 | 0.11 | 13.30 | 0.000 | 3.33 | 5.05 |
| *Group: Eco Position* | 0.76 | 0.11 | -2.58 | 0.010 | 0.61 | 0.93 |
| *Group: Eco Position & Labels* | 0.70 | 0.11 | -3.35 | 0.001 | 0.56 | 0.86 |
| *Gender: Male* | 0.98 | 0.03 | -0.69 | 0.490 | 0.92 | 1.04 |
| *Age: 18-24* | 0.84 | 0.04 | -4.63 | 0.000 | 0.78 | 0.90 |
| *Age: 35-54* | 1.01 | 0.04 | 0.15 | 0.884 | 0.94 | 1.08 |
| *Income: £0-25K* | 1.07 | 0.03 | 1.89 | 0.058 | 1.00 | 1.14 |
| *Income: Prefer not to say* | 0.99 | 0.07 | -0.19 | 0.849 | 0.85 | 1.14 |
| *Education: Lower* | 1.10 | 0.03 | 3.19 | 0.001 | 1.04 | 1.17 |
| *Meat consumption: Medium* | 1.08 | 0.12 | 0.67 | 0.505 | 0.85 | 1.38 |
| *Meat consumption: Low* | 0.90 | 0.14 | -0.77 | 0.442 | 0.68 | 1.18 |
| *Group: Eco Position * Meat consumption: Medium* | 1.03 | 0.13 | 0.24 | 0.814 | 0.80 | 1.34 |
| *Group: Eco Position & Labels * Meat consumption: Medium* | 1.03 | 0.13 | 0.21 | 0.836 | 0.79 | 1.33 |
| *Group: Eco Position * Meat consumption: Low* | 1.04 | 0.15 | 0.25 | 0.801 | 0.77 | 1.40 |
| *Group: Eco Position & Labels * Meat consumption: Low* | 1.05 | 0.15 | 0.34 | 0.732 | 0.78 | 1.42 |

*Note.* Intercepts, coefficients and confidence intervals were exponentiated to facilitate interpretation of the log-transformed dependent variable (mean ecoscore of shopping baskets).

# Appendix H. Interaction models for a) health and b) environmental consciousness

|  | **Estimate** | **Std. error** | **Statistic** | **p-value** | **95% Conf. low** | **95% Conf. high** |
| --- | --- | --- | --- | --- | --- | --- |
| 1. **Health** | | | | | | |
| *Intercept* | 36.01 | 0.90 | 39.88 | 0.000 | 34.24 | 37.79 |
| *Health Position* | -1.60 | 1.02 | -1.57 | 0.117 | -3.60 | 0.40 |
| *Health Position & Labels* | -1.47 | 1.04 | -1.41 | 0.158 | -3.51 | 0.57 |
| *Health consciousness: Moderately important* | -0.72 | 1.12 | -0.64 | 0.523 | -2.92 | 1.49 |
| *Health consciousness: Very important/important* | -0.18 | 1.02 | -0.18 | 0.859 | -2.19 | 1.83 |
| *Health Position * Moderately important* | 0.01 | 1.26 | 0.01 | 0.993 | -2.45 | 2.48 |
| *Health Position & Labels * Moderately important* | -0.28 | 1.27 | -0.22 | 0.826 | -2.78 | 2.22 |
| *Health Position * Very important/important* | -1.15 | 1.15 | -1.00 | 0.317 | -3.40 | 1.10 |
| *Health Position & Labels * Very important/important* | -1.55 | 1.16 | -1.33 | 0.183 | -3.84 | 0.73 |
| 1. **Eco** |  |  |  |  |  |  |
| *Intercept* | 4.37 | 0.08 | 17.36 | 0.000 | 3.70 | 5.16 |
| *Eco Position* | 0.83 | 0.09 | -1.99 | 0.047 | 0.70 | 1.00 |
| *Eco Position & Labels* | 0.78 | 0.09 | -2.73 | 0.006 | 0.65 | 0.93 |
| *Eco consciousness: Moderately important* | 1.02 | 0.11 | 0.15 | 0.884 | 0.82 | 1.27 |
| *Eco consciousness: Very important/important* | 0.83 | 0.13 | -1.47 | 0.141 | 0.64 | 1.07 |
| *Eco Position * Moderately important* | 0.86 | 0.12 | -1.24 | 0.215 | 0.67 | 1.09 |
| *Eco Position & Labels * Moderately important* | 0.86 | 0.12 | -1.18 | 0.240 | 0.68 | 1.10 |
| *Eco Position * Very important/important* | 0.92 | 0.14 | -0.59 | 0.557 | 0.70 | 1.21 |
| *Eco Position & Labels * Very important/important* | 0.90 | 0.14 | -0.75 | 0.456 | 0.68 | 1.19 |

#

*Note.* Intercepts, coefficients and confidence intervals of the eco models were exponentiated to facilitate interpretation.

# Appendix I. Adjusted models comparing positioning & label groups to positioning only

|  | **Estimate** | **Standard error** | **Statistic** | **p-value** | **95% conf. interval low** | **95% conf. interval high** |
| --- | --- | --- | --- | --- | --- | --- |
| 1. **Adjusted health label model – Main analysis** | | | | | | |
| *Intercept* | 34.99 | 0.51 | 68.42 | 0.000 | 33.99 | 36.00 |
| *Group: Health Position & Labels* | -0.19 | 0.23 | -0.84 | 0.404 | -0.65 | 0.26 |
| *Gender: Male* | -0.06 | 0.24 | -0.26 | 0.793 | -0.52 | 0.40 |
| *Age: 18-24* | -0.79 | 0.30 | -2.60 | 0.009 | -1.38 | -0.19 |
| *Age: 35-54* | -0.18 | 0.28 | -0.65 | 0.516 | -0.72 | 0.36 |
| *Income: £0-25K* | -0.07 | 0.24 | -0.27 | 0.788 | -0.55 | 0.41 |
| *Income: Prefer not to say* | 0.70 | 0.26 | 2.69 | 0.007 | 0.19 | 1.22 |
| *Education: Lower* | 1.19 | 0.58 | 2.05 | 0.040 | 0.05 | 2.33 |
| *Meat consumption: Medium* | -0.52 | 0.29 | -1.81 | 0.070 | -1.08 | 0.04 |
| *Meat consumption: Low* | -0.59 | 0.33 | -1.79 | 0.073 | -1.23 | 0.06 |
| *Health consciousness: Moderately important* | -0.82 | 0.41 | -1.99 | 0.047 | -1.63 | -0.01 |
| *Health consciousness: Very important/important* | -1.47 | 0.39 | -3.81 | 0.000 | -2.23 | -0.72 |
| 1. **Adjusted health label model – Sensitivity analysis** | | | | | | |
| *Intercept* | 34.79 | 0.65 | 53.35 | 0.000 | 33.51 | 36.08 |
| *Group: Health Position & Labels* | -0.67 | 0.30 | -2.25 | 0.025 | -1.26 | -0.09 |
| *Gender: Male* | 0.12 | 0.31 | 0.41 | 0.683 | -0.47 | 0.72 |
| *Age: 18-24* | -1.15 | 0.39 | -2.95 | 0.003 | -1.91 | -0.38 |
| *Age: 35-54* | -0.12 | 0.36 | -0.34 | 0.732 | -0.84 | 0.59 |
| *Income: £0-25K* | 0.09 | 0.32 | 0.28 | 0.781 | -0.54 | 0.71 |
| *Income: Prefer not to say* | 0.44 | 0.33 | 1.32 | 0.186 | -0.21 | 1.10 |
| *Education: Lower* | 1.47 | 0.71 | 2.08 | 0.038 | 0.08 | 2.86 |
| *Meat consumption: Medium* | -0.37 | 0.38 | -0.99 | 0.321 | -1.11 | 0.36 |
| *Meat consumption: Low* | -0.94 | 0.44 | -2.15 | 0.032 | -1.81 | -0.08 |
| *Health consciousness: Moderately important* | -0.16 | 0.52 | -0.31 | 0.756 | -1.19 | 0.87 |
| *Health consciousness: Very important/important* | -1.02 | 0.50 | -2.02 | 0.044 | -2.00 | -0.03 |
| 1. **Adjusted eco label model – Main analysis** | | | | | | |
| *Intercept* | 3.51 | 0.05 | 24.76 | 0.000 | 3.18 | 3.88 |
| *Group: Eco Position & Labels* | 0.93 | 0.03 | -2.34 | 0.020 | 0.88 | 0.99 |
| *Gender: Male* | 0.95 | 0.03 | -1.70 | 0.090 | 0.89 | 1.01 |
| *Age: 18-24* | 0.88 | 0.04 | -3.27 | 0.001 | 0.81 | 0.95 |
| *Age: 35-54* | 1.02 | 0.04 | 0.59 | 0.557 | 0.95 | 1.10 |
| *Income: £0-25K* | 1.09 | 0.03 | 2.79 | 0.005 | 1.03 | 1.17 |
| *Income: Prefer not to say* | 1.05 | 0.03 | 1.48 | 0.138 | 0.98 | 1.13 |
| *Education: Lower* | 0.97 | 0.08 | -0.45 | 0.651 | 0.83 | 1.12 |
| *Meat consumption: Medium* | 1.12 | 0.04 | 3.00 | 0.003 | 1.04 | 1.20 |
| *Meat consumption: Low* | 0.95 | 0.04 | -1.11 | 0.267 | 0.88 | 1.04 |
| *Environmental consciousness: Moderately important* | 0.87 | 0.04 | -3.79 | 0.000 | 0.81 | 0.93 |
| *Environmental consciousness: Very important/important* | 0.77 | 0.04 | -7.12 | 0.000 | 0.71 | 0.83 |
| 1. **Adjusted eco label model – Sensitivity analysis** | | | | | | |
| *Intercept* | 3.80 | 0.07 | 19.17 | 0.000 | 3.32 | 4.36 |
| *Group: Eco Position & Labels* | 0.93 | 0.04 | -1.74 | 0.083 | 0.85 | 1.01 |
| *Gender: Male* | 0.97 | 0.05 | -0.67 | 0.503 | 0.89 | 1.06 |
| *Age: 18-24* | 0.88 | 0.06 | -2.28 | 0.023 | 0.78 | 0.98 |
| *Age: 35-54* | 1.10 | 0.05 | 1.88 | 0.061 | 1.00 | 1.22 |
| *Income: £0-25K* | 1.09 | 0.05 | 1.83 | 0.068 | 0.99 | 1.19 |
| *Income: Prefer not to say* | 1.03 | 0.05 | 0.66 | 0.512 | 0.94 | 1.14 |
| *Education: Lower* | 0.80 | 0.11 | -2.07 | 0.039 | 0.64 | 0.99 |
| *Meat consumption: Medium* | 1.03 | 0.05 | 0.58 | 0.562 | 0.93 | 1.14 |
| *Meat consumption: Low* | 0.93 | 0.06 | -1.23 | 0.221 | 0.82 | 1.05 |
| *Environmental consciousness: Moderately important* | 0.84 | 0.05 | -3.33 | 0.001 | 0.75 | 0.93 |
| *Environmental consciousness: Very important/important* | 0.74 | 0.05 | -5.62 | 0.000 | 0.67 | 0.82 |

*Note.* Intercepts, coefficients and confidence intervals of the eco models were exponentiated to facilitate interpretation.

# Appendix J. Means and standard deviations for secondary outcomes by study condition

| **Mean Energy Density** |  |
| --- | --- |
| Control | 220.12 (20.60) |
| Health Position | 214.40 (21.65) |
| Health Position + Labelling | 213.05 (23.14) |
| Sustainability Position | 222.19 (23.04) |
| Sustainability Position + Labelling | 222.97 (21.67) |
| **Mean Salt Content** |  |
| Control | .77 (.15) |
| Health Position | .73 (.11) |
| Health Position + Labelling | .71 (.12) |
| Sustainability Position | .80 (.21) |
| Sustainability Position + Labelling | .80 (.12) |
| **Mean Fat Content** |  |
| Control | 10.55 (2.11) |
| Health Position | 9.88 (1.95) |
| Health Position + Labelling | 9.78 (2.16) |
| Sustainability Position | 10.93 (2.02) |
| Sustainability Position + Labelling | 10.98 (1.86) |
| **Mean Sugar Content** |  |
| Control | 4.89 (1.10) |
| Health Position | 4.91 (1.26) |
| Health Position + Labelling | 4.83 (1.26) |
| Sustainability Position | 4.96 (1.51) |
| Sustainability Position + Labelling | 4.85 (1.27) |
| **Mean Greenhouse Gas Emissions** |  |
| Control | .55 (.26) |
| Health Position | .54 (.23) |
| Health Position + Labelling | .56 (.26) |
| Sustainability Position | .43 (.23) |
| Sustainability Position + Labelling | .39 (.23) |
| **Mean Land Use** |  |
| Control | .81 (.54) |
| Health Position | .80 (.55) |
| Health Position + Labelling | .85 (.63) |
| Sustainability Position | .64 (.48) |
| Sustainability Position + Labelling | .60 (.47) |
| **Mean Water Use** |  |
| Control | 1520.82 (461.95) |
| Health Position | 1411.57 (405.92) |
| Health Position + Labelling | 1418.54 (411.31) |
| Sustainability Position | 1159.93 (420.48) |
| Sustainability Position + Labelling | 1100.59 (418.84) |
| **Mean Eutrophication** |  |
| Control | 3.25 (2.35) |
| Health Position | 3.25 (2.44) |
| Health Position + Labelling | 3.35 (2.43) |
| Sustainability Position | 2.71 (2.38) |
| Sustainability Position + Labelling | 2.45 (2.19) |
| **Mean Basket Price** |  |
| Control | 24.09 (5.56) |
| Health Position | 22.78 (5.20) |
| Health Position + Labelling | 22.45 (5.34) |
| Sustainability Position | 22.48 (4.76) |
| Sustainability Position + Labelling | 22.33 (4.81) |

# Appendix K. Tukey test of price for sustainability intervention groups and control, with outliers removed

|  | **Estimate** | **95% Conf. low** | **95% Conf. high** | **p-value** |
| --- | --- | --- | --- | --- |
| 1. **Health** | | | | |
| *Control vs Sustainability Position* | -0.95 | -1.97 | 0.07 | 0.074 |
| *Control vs. Sustainability Position & Labels* | -1.07 | -2.09 | -0.05 | 0.037 |
| *Sustainability Position vs. Sustainability Position & Labels* | -0.12 | -0.70 | 0.46 | 0.878 |

Significance threshold of p<0.025.

# Appendix L. Impact of eco position and eco position & labels interventions on mean Nutri-Score of products in shopping baskets, model with outliers removed

|  | **Estimate** | **Std. error** | **Statistic** | **p-value** | **95% Conf. low** | **95% Conf. high** |
| --- | --- | --- | --- | --- | --- | --- |
| 1. **Eco** |  |  |  |  |  |  |
| *Control (ref)* | 35.88 | 0.33 | 108.43 | 0.000 | 35.23 | 36.53 |
| *Eco Position* | 1.07 | 0.36 | 2.94 | 0.003 | 0.35 | 1.78 |
| *Eco Position & Labels* | 0.70 | 0.36 | 1.92 | 0.055 | -0.01 | 1.41 |
